# Pan-cancer analysis of *NUP155* and validation of its role in breast cancer cell proliferation, migration, and apoptosis

**DOI:** 10.1186/s12885-024-12039-6

**Published:** 2024-03-19

**Authors:** Zi-qiong Wang, Zhi-xuan Wu, Zong-pan Wang, Jing-xia Bao, Hao-dong Wu, Di-yan Xu, Hong-feng Li, Yi-Yin Xu, Rong-xing Wu, Xuan-xuan Dai

**Affiliations:** 1 Quzhou People’s Hospital, The Quzhou Affiliated Hospital of Wenzhou Medical University, 100 Minjiang Avenue, Quzhou, Zhejiang, 324000 Zhejiang China; 2 Department of Breast Surgery, The First Affiliated Hospital of Wenzhou Medical University, Wenzhou, 325035 Zhejiang China; 3 Key Laboratory of Clinical Laboratory Diagnostics (Ministry of Education), The First Affiliated Hospital of Wenzhou Medical University, Wenzhou, 325035 China

**Keywords:** *NUP155*, Pan-cancer analysis, Prognosis, Immune infiltration, Immune checkpoints, Molecular biology experiments

## Abstract

*NUP155* is reported to be correlated with tumor development. However, the role of *NUP155* in tumor physiology and the tumor immune microenvironment (TIME) has not been previously examined. This study comprehensively investigated the expression, immunological function, and prognostic significance of *NUP155* in different cancer types. Bioinformatics analysis revealed that *NUP155* was upregulated in 26 types of cancer. Additionally, *NUP155* upregulation was strongly correlated with advanced pathological or clinical stages and poor prognosis in several cancers. Furthermore, *NUP155* was significantly and positively correlated with DNA methylation, tumor mutational burden, microsatellite instability, and stemness score in most cancers. Additionally, *NUP155* was also found to be involved in TIME and closely associated with tumor infiltrating immune cells and immunoregulation-related genes. Functional enrichment analysis revealed a strong correlation between *NUP155* and immunomodulatory pathways, especially antigen processing and presentation. The role of *NUP155* in breast cancer has not been examined. This study, for the first time, demonstrated that *NUP155* was upregulated in breast invasive carcinoma (BRCA) cells and revealed its oncogenic role in BRCA using molecular biology experiments. Thus, our study highlights the potential value of *NUP155* as a biomarker in the assessment of prognostic prediction, tumor microenvironment and immunotherapeutic response in pan-cancer.

**Supplementary Information:**

The online version contains supplementary material available at 10.1186/s12885-024-12039-6.

## Introduction

Cancer adversely affects human health and quality of life worldwide. In addition to the number of newly diagnosed cancer cases, the burden of cancer is increasing due to rapid population aging [1, 2]. The breakthrough in immune checkpoint inhibitor (ICI) therapy has enabled the development of immunotherapy, which is a novel therapeutic approach that improves the clinical outcomes of patients with cancer [3, 4]. Therefore, there is a need to explore novel immunotherapeutic targets and their roles in tumor physiology and tumor immune microenvironment (TIME).

The nuclear pore complex (NPC), a specific protein complex for transmembrane transport, functions as a channel for importing and exporting nuclear molecules [5–7]. Dysfunctional NPC can lead to various diseases, including cancer [6, 8]. Nucleoproteins, which are the structural components of the NPC, regulate the progression of cancer through the following three main mechanisms: modulation of protein expression levels, induction of chromosomal translocations that result in the generation of fusion proteins, and induction of single point mutations [9, 10]. Various cancer cells, especially multidrug-resistant and aggressive tumor cells, exhibit upregulated levels of nuclear proteins, high rates of nucleoplasmic translocation, and dependency on the nuclear translocation system. This indicates that the nuclear translocation machinery can be a potential therapeutic target for cancer [11]. Additionally, nucleoplasmic transport inhibitors have been subjected to partial clinical trials as they are reported to effectively induce cancer cell death [12, 13]. *NUP155* is actively involved in nuclear pore formation, as well as in selective gene regulation in pathological conditions [14–16]. Besides, a previously published study demonstrated that *NUP155* mutations can result in specific phenotypes associated with atrial fibrillation in mice and humans [17]. Recent studies have reported that *NUP155* expression is correlated with the prognosis of various cancers [18, 19]. Additionally, *NUP155* activates the cell cycle protein-dependent kinase inhibitor p21 in the p53 (tumor suppressor) pathway and has a key role in the transcriptional response to DNA damage [20, 21]. Basit et al. demonstrated that the cGAS-STING-TBK1-IRF3 signaling-mediated regulation of p21 in the innate immune response affected chromosomal stability [22]. Thus, there is growing evidence linking NUP155 to tumor development. However, previous studies have not examined the role of *NUP155* in tumor physiology and TIME in pan-cancer datasets.

This study aimed to comprehensively analyze the expression pattern, prognostic value, and immunological functions of *NUP155* across 33 types of cancer. The correlation of *NUP155* expression with DNA promoter methylation, somatic mutations, tumor mutational burden (TMB), microsatellite instability (MSI), tumor stemness, mismatch repair (MMR), TIME, infiltrating immune cell profile, and immune-related biomarkers was further investigated. Additionally, single-cell RNA sequencing dataset and immunotherapy cohort data analyses indicated that *NUP155* is a potential biomarker for predicting the efficacy of immunotherapy. Furthermore, the oncogenic role of *NUP155* in breast invasive carcinoma (BRCA) was validated using molecular biology experiments.

## Methods

### Data collection

The RNA sequencing and clinical data were downloaded from TCGA and GTEx databases with the UCSC Xena browser [23]. The expression data of tumor cell lines and tissues downloaded from the CCLE database were analyzed according to tissue origin. The UALCAN database [24] was used to examine the DNA methylation and protein levels of NUP155 between cancer and corresponding normal tissues. Tumor Immunology Single Cell Center (TISCH) [25], a single-cell RNA (scRNA) sequencing database of gene expression levels in the TIME, was used for characterizing *NUP155* expression profiles in the microenvironment at the single-cell level. The response to immunotherapy was examined using two immunotherapy cohorts (GSE78220 cohort: patients with melanoma; Imvigor210 cohort: patients with metastatic uroepithelial carcinoma).

### Pathological or clinical stage and prognosis

*NUP155* expression in TCGA dataset was investigated at different pathological or clinical stages of pan-cancer using statistical methods, including Kruskal-Wallis Test and Dunn’s test [26–28]. When the data comprised < 3 samples or the standard deviation of the data was 0, stages I and II were combined for early-stage tumors or stages III and IV were combined for late-stage tumors before performing statistical analysis. The prognostic significance of *NUP155* was examined using the univariate Cox proportional hazard model and Kaplan-Meier (KM) survival analysis with “survminer” R package. The best cut-off scores were used to determine the overall survival (OS), disease-specific survival (DSS), and progression-free survival (PFS) in the high-expression and low-expression cohorts.

### TMB, MSI, and MMR analyses

The Simple Nucleotide Variation dataset of all TCGA samples processed using MuTect2 software was downloaded from Genomics Data Commons (GDC) [29]. The TMB for each tumor was determined using the “maftools” R package. Additionally, the MSI score was obtained from a previous study [30]. The expression level of MMR genes was assessed based on the expression profile data from TCGA [31, 32].

### Somatic mutation and stemness score analyses

The cBioPortal website [33, 34] was used to analyze the correlation between *NUP155* expression and somatic mutations among pan-cancer. To investigate the correlation between *NUP155* expression and tumor stemness score, the gene expression data obtained from previous studies were integrated with the stemness score of each tumor, and the methylation feature was calculated.

### Immune cell infiltration and immune modulator gene analyses

The immune and stromal fraction scores for various tumor samples were determined using the ESTIMATE algorithm. The correlation between *NUP155* expression and the immune and stromal fraction scores was determined using the ‘estimate’ and ‘limma’ R packages. For reliable immune score assessment, xCell and CIBERSORT analyses were performed using the ‘IOBR’ R package. Next, co-expression analysis of *NUP155* and immunoregulation-related genes was performed.

### Drug sensitivity analysis

The correlation between *NUP155* expression and drug sensitivity was analyzed using the Genomics of Drug Sensitivity in Cancer (GDSC) and Cancer Therapeutics Response Portal (CTRP) databases with the Gene Set Cancer Analysis (GSCA) platform [35]. Additionally, the correlation between *NUP155* expression and sensitivity to 263 drugs approved by the Food and Drug Administration or undergoing clinical trials was examined using the CellMiner (NCI-60) database.

### Construction of protein-protein interaction network (PPI) and functional annotation

GeneMANIA [36], which is a website designed to build PPI networks, provides gene function prediction hypotheses and identifies comparable genes. In this study, the PPI network for NUP155 was constructed using GeneMANIA to explore the interactions between NUP155 and NUP155-related genes.

The biological function of *NUP155* in pan-cancer was examined using gene set enrichment analysis (GSEA). The gene sets of Gene Ontology (GO), Kyoto Encyclopedia of Genes and Genomes (KEGG), and REACTOME were downloaded from the GSEA website. The top 100 co-expressed genes were mapped using the R package ‘clusterProfiler’ for enrichment analysis.

### Cell culture and quantitative real-time polymerase chain reaction (qRT-PCR) and western blotting analyses

Normal human breast cells (MCF-10 A cells) and breast cancer cell lines (BT-549, MDA-MB-231, and T-47D cells) were purchased from the National Collection of Authenticated Cell Cultures. The cells were cultured in a humidified atmosphere containing 5% CO_2_. The culture medium was regularly replaced until the cells achieved 80–90% confluency. The primer sequences for the human target gene *NUP155* that were purchased from Biosepur were as follows: 5′-CTTAGTGTCTACCTGGCTGCTTGG-3′ (forward primer); 5′-TGATGCTGATGCTGATGCTTCTGG-3′ (reverse primer). Total RNA was extracted from the four cell lines using an RNA extraction kit (Takara). The extracted RNA was then reverse-transcribed to complementary DNA using the reverse transcription kit (Beyotime). qRT-PCR analysis was performed using an Exicycler 96 instrument (BIONEER). The expression levels of *NUP155* were normalized to those of *GAPDH*. The relative expression levels of the target gene were calculated using the ΔΔCq method [37].

The small interfering RNA (siRNA) oligonucleotides targeting *NUP155* (si-NUP155) and scrambled siRNA were designed and synthesized by General Biol Corporation (Anhui, China). The cells were transfected with si-NUP155, washed thrice with phosphate-buffered saline (PBS), and harvested using centrifugation. Total proteins were extracted using radioimmunoprecipitation assay buffer supplemented with a protease inhibitor cocktail (R0010, Solarbio). Western blotting analysis was performed using anti-NUP155 (66359-1-Ig, Proteintech), anti-ACTB (66009-1-Ig, Proteintech), anti-BAX (50599-2-Ig, Proteintech), and anti-BCL2 (68103-1-Ig, Proteintech) antibodies, following the manufacturer’s instructions. The blots were cut prior to hybridisation with antibodies during blotting. The secondary antibodies used in this study were horseradish peroxidase (HRP)-conjugated goat anti-mouse IgG (SA00001-1, Proteintech) or HRP-conjugated goat anti-rabbit IgG (SA00001-2, Proteintech). ACTB was used as a loading control. Immunoreactive signals were developed using an enhanced chemiluminescence reagent (4 A Biotech, China).

### Cell viability assay

Cells were seeded at a density of 2000 cells/well in 96-well plates and cultured for 0, 24, 48, and 72 h. Next, the cells were incubated with 10 µL of cell counting kit-8 (CCK-8) solution for 120 min. The absorbance of the sample at 450 nm was measured.

### Transwell assay

Cells were seeded in the upper chamber containing serum-free medium at a density of 2 × 10^6^ cells/well. In the lower chamber, 500 µL of medium containing 20% fetal bovine serum was added. After incubation at room temperature and 5% (v/v) CO_2_ for 24 h, non-invasive cells in the upper chamber were removed. Meanwhile, the cells on the bottom surface were fixed using a 10% neutral buffered formalin solution and stained with 0.1% crystal violet. The invasive cells were counted in five randomly selected microscopic fields.

### Wound healing assay

After treating logarithmic growth phase cells from the third to the fifth passage, the cells were seeded in six-well plates at a density of 1 × 10^6^ cells/mL and cultured for 24 h in a CO_2_ incubator until they reached approximately 70% confluency. A sterile pipette tip was used to gently generate a horizontal scratch in the monolayer. The cells were gently washed thrice with PBS to remove detached cells. Next, the cells were cultured in a serum-free medium for 24 h in a CO_2_ incubator and fixed using a methanol solution. The closure of the cell scratch was monitored using an inverted microscope after crystal violet staining.

### Statistical analyses

All statistical analyses were performed using R software (version 4.0.2) and GraphPad Prism 7. As the gene expression levels exhibited highly right-skewed distribution in TCGA dataset, the gene expression data were normalized using log-2 transformation (X to Log2(X + 1)). Survival was analyzed using Cox regression analysis, the KM method, and log-rank tests. The correlation between two variables was analyzed using Spearman or Pearson tests. To analyze the molecular biology experiment data, means between two groups were compared using the two-tailed Student’s t-test. Data are expressed as mean ± standard error of the mean. Differences were considered significant at *P* < 0.05. The R-scripts and online tools used in this study are shown in Supplementary Table S1**.**

## Results

### Differential expression ofNUP155 between normal and cancer tissues

**Fig. 1:**
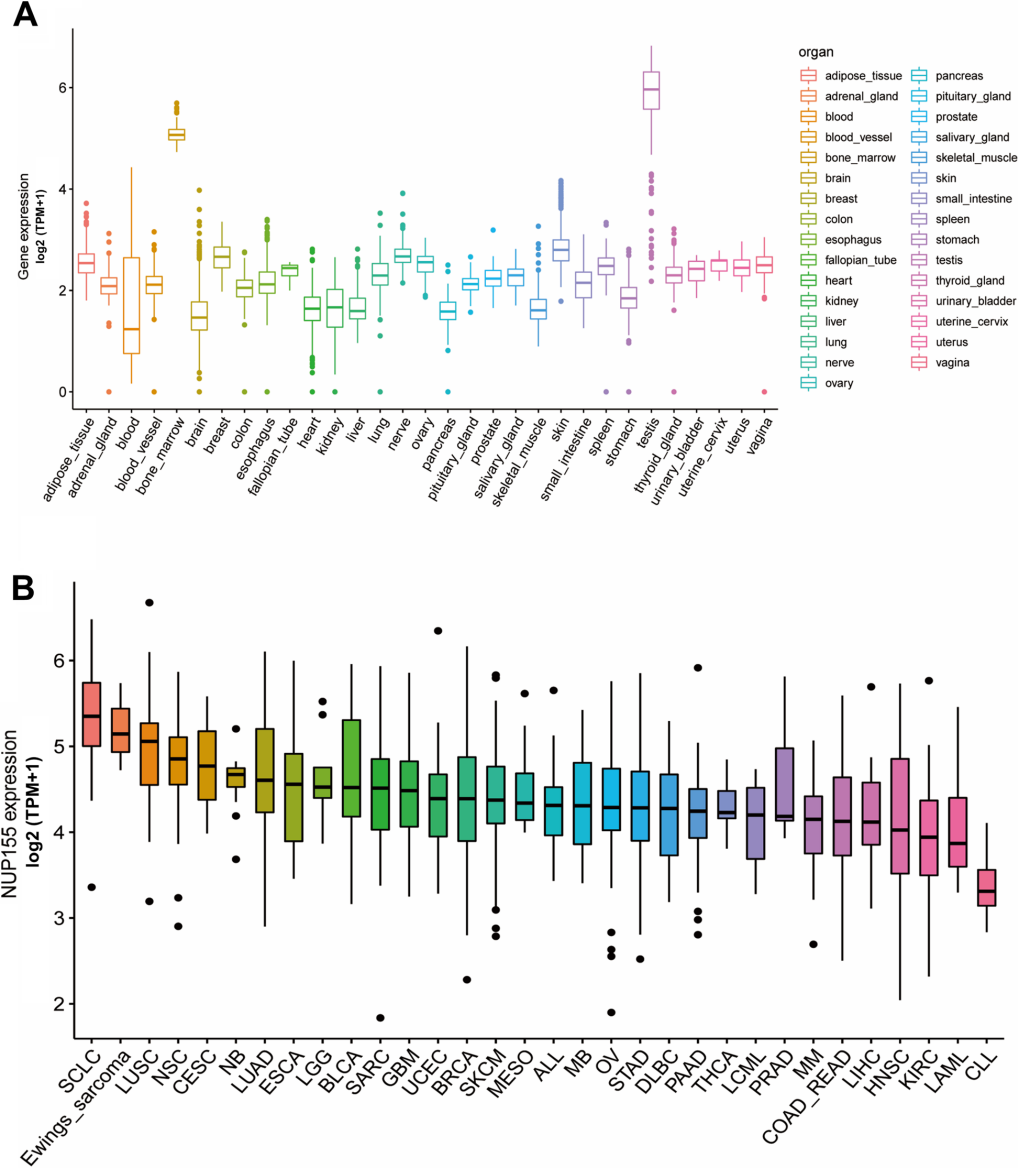
Differential expression of *NUP155* in pan-cancer. **A** *NUP155* expression in normal tissues. **B** *NUP155* expression in tumor cell lines

**Fig. 2:**
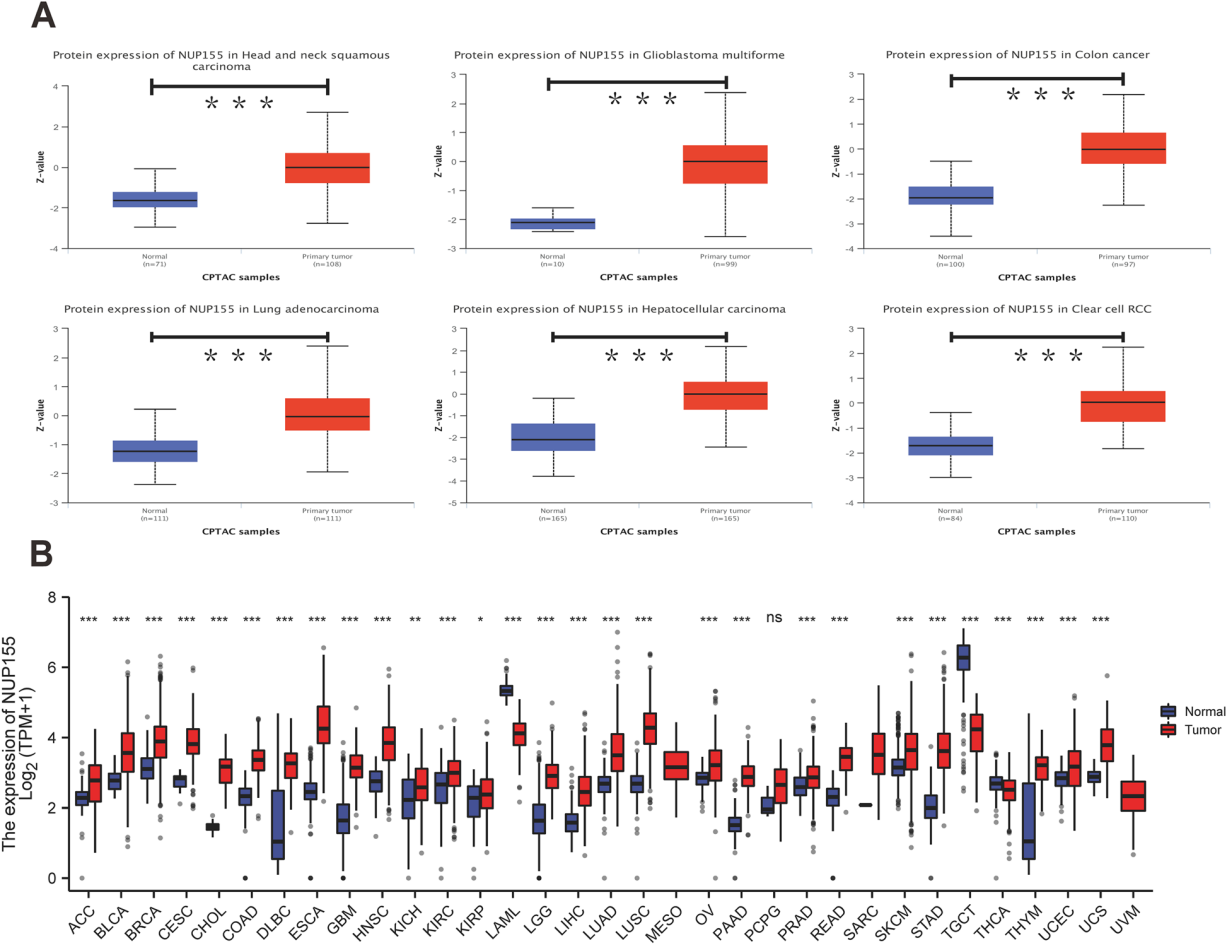
Differential expression of *NUP155* in pan-cancer. **A** NUP155 protein expression level in normal tissues and primary tissues of HNSC, GBM, COAD, LUAD, HCC and RCC. **B** Comparison of *NUP155* expression between tumor and normal tissues. *p < 0.05; **p < 0.01; ***p < 0.001

Analysis of GTEx datasets revealed that the mRNA expression levels of *NUP155* were comparable in all organs, except bone marrow and testis **(**Fig. 1A**)**. The *NUP155* expression levels were downregulated in most healthy tissues. Figure 1B shows the relative expression levels of *NUP155* in different cell lines in the CCLE dataset. The *NUP155* expression levels varied in different cancer cell lines with the small cell lung cancer cell line exhibiting upregulated expression levels. Analysis of NUP155 protein expression using the UALCAN database revealed that the NUP155 expression levels in head and neck squamous cell carcinoma (HNSC), glioblastoma multiforme (GBM), colon cancer, lung adenocarcinoma (LUAD), hepatocellular carcinoma (HCC), and clear cell renal cell carcinoma (RCC) were significantly upregulated when compared with those in the corresponding non-cancerous tissues **(**Fig. 2A**)**. The *NUP155* mRNA expression levels varied between tumor and non-cancerous tissue in 29 cancers (samples for which non-cancerous tissue data were not available were excluded) **(**Fig. 2B**)**. Compared with those in non-cancerous tissues, the *NUP155* expression levels were upregulated in adrenocortical carcinoma (ACC), bladder urothelial carcinoma (BLCA), BRCA, cervical squamous cell carcinoma and endocervical adeno carcinoma (CESC), cholangiocarcinoma (CHOL), colon adenocarcinoma (COAD), lymphoid neoplasm diffuse large B-cell lymphoma (DLBC), esophageal carcinoma (ESCA), GBM, HNSC, kidney chromophobe (KICH), kidney renal clear cell carcinoma (KIRC), kidney renal papillary cell carcinoma (KIRP), brain lower grade glioma (LGG), liver hepatocellular carcinoma (LIHC), LUAD, lung squamous cell carcinoma (LUSC), ovarian serous cystadenocarcinoma (OV), pancreatic adenocarcinoma (PAAD), prostate adenocarcinoma (PRAD), rectum adenocarcinoma (READ), skin cutaneous melanoma (SKCM), stomach adenocarcinoma (STAD), thymoma (THYM), uterine corpus endometrial carcinoma (UCEC), and uterine carcinosarcoma (UCS) tissues. In contrast, the *NUP155* expression levels in acute myeloid leukemia (LAML), testicular germ cell tumor (TGCT), and thyroid carcinoma (THCA) tissues were downregulated when compared with those in non-cancerous tissues. The differential expression of *NUP155* between cancer and non-cancerous tissues was the most pronounced in DLBC and THYM. However, *NUP155* expression was not significantly different between cancer and non-cancerous tissues in mesothelioma (MESO), pheochromocytoma and paraganglioma (PCPG), and sarcoma (SARC).

### Differential expression of *NUP155* between normal and cancer tissues

*NUP155* expression was significantly correlated with pathological or clinical stage in ACC, KICH, KIRC, KIRP, LIHC, OV, SKCM, and UCS (Supplementary Fig. 1). In particular, *NUP155* expression was positively correlated with advanced tumor stage in ACC, KICH, KIRP, and LIHC.

### Methylation profile and genetic alterations of *NUP155*

**Fig. 3:**
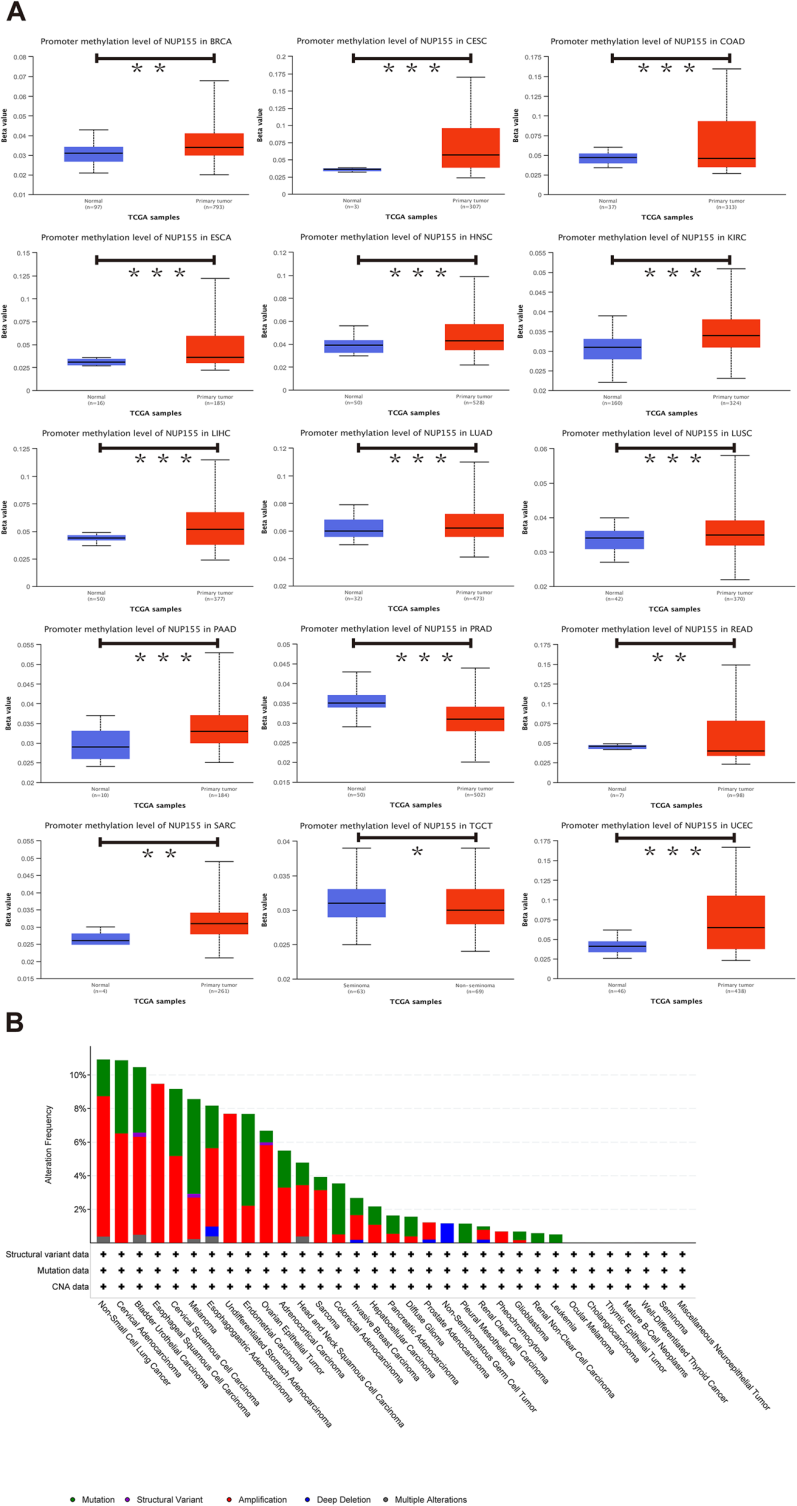
DNA methylation and mutation features of *NUP155* in pan-cancer. **A** Promoter methylation level of *NUP155*. **B** The alteration frequency and different mutation types of *NUP155*. *p < 0.05; **p < 0.01; ***p < 0.001

DNA methylation alterations in cancer are powerful diagnostic and prognostic targets. Analysis of the UALCAN dataset revealed that compared with those in non-cancerous tissues, the methylation levels of *NUP155* were upregulated in BRCA, CESC, ESCA, HNSC, KIRC, LIHC, LUAD, LUSC, PAAD, SARC, and UCEC tissues and downregulated in COAD, PRAD, READ, and TGCT tissues **(**Fig. 3A and Supplementary Table S2**)**. The cBioPortal database was used to investigate the *NUP155* alterations in pan-cancer. The frequency of *NUP155* alterations was the highest in non-small cell lung cancer (approximately 10%) **(**Fig. 3B**)**. Amplifications and mutations were the most frequent genetic alterations.

Furthermore, the landscape of *NUP155* copy number variation (CNV) in pan-cancer was examined. This study analyzed the correlation between *NUP155* CNV and *NUP155* mRNA levels using the GSCA online website. The *NUP155* methylation levels were closely associated with *NUP155* mRNA expression levels in various cancer types, including LUSC, LUAD, HNSC, SARC, BLCA, OV, BRCA, ESCA, CESC, STAD, SKCM, UCS, KIRC, KICH, COAD, LIHC, UCEC, KIRP, PCPG, READ, and LGG (Supplementary Fig. 2 and Supplementary Table S3).

### Prognostic value of *NUP155* expression in different cancer types

**Fig. 4:**
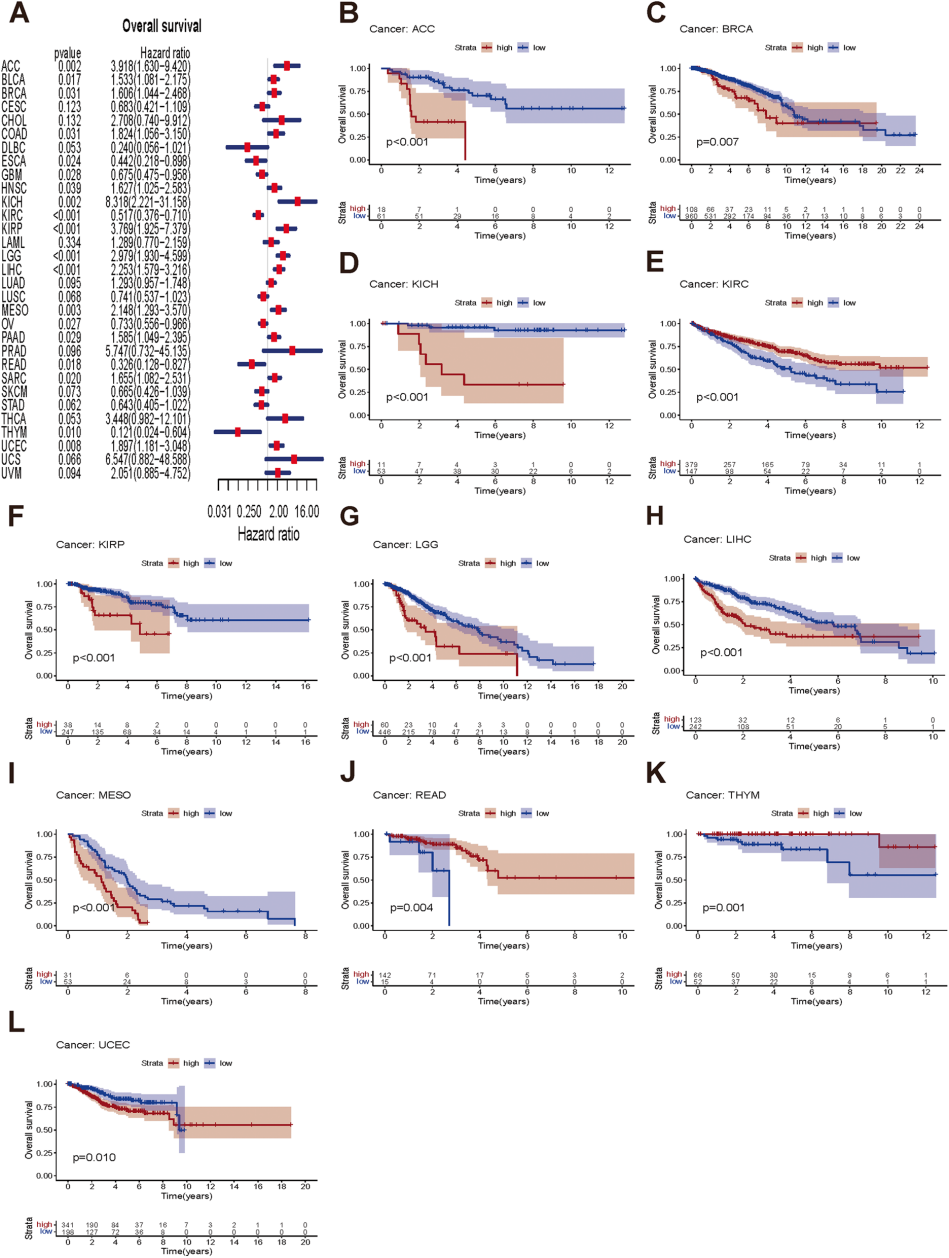
Association between *NUP155* expression levels and OS in TCGA pan-cancer. **A** Forest plot of association of *NUP155* expression and OS. **B**–**L** Kaplan-Meier analysis of the association between *NUP155* expression and OS.

Next, this study examined the prognostic value of *NUP155* in different cancer types using survival analyses. The three endpoints of this study were OS, DSS, and PFS. *NUP155* expression was significantly correlated with OS in the following 19 types of cancer: ACC, BLCA, BRCA, COAD, ESCA, GBM, HNSC, KICH, KIRC, KIRP, LGG, LIHC, MESO, OV, PAAD, READ, SARC, THYM, and UCEC **(**Fig. 4A**)**. The KM survival curves revealed that *NUP155* upregulation was significantly correlated with poor OS in ACC, BRCA, KICH, KIRP, LGG, LIHC, MESO, and UCEC and favorable OS in KIRC, READ, and THYM **(**Fig. 4B–L**)**. *NUP155* expression was correlated with DSS in the following nine types of cancer: ACC, KICH, KIRC, KIRP, LGG, LIHC, MESO, THYM, and UCEC (Supplementary Fig. 3A). The KM survival curves indicated that *NUP155* upregulation was associated with poor DSS in ACC, KICH, KIRP, LGG, LIHC, MESO, and UCEC and favorable DSS in KIRC, THYM, and UCEC (Supplementary Fig. 3B–J). Additionally, the effect of *NUP155* dysregulation on PFS was investigated (Supplementary Fig. 4A). Univariate Cox regression analysis revealed that *NUP155* expression is a risk factor for PFS in ACC, BRCA, KICH, KIRP, LGG, LIHC, MESO, UCEC, and uveal melanoma (UVM) and an active factor for PFS in KIRC (Supplementary Fig. 4B–K). KM analysis suggested that *NUP155* upregulation was associated with unfavorable PFS in patients with ACC, BLCA, KICH, KIRP, LIHC, LGG, MESO, UCEC, and UVM and favorable PFS in patients with KIRC. Thus, *NUP155* upregulation is associated with poor prognosis in most cancers.

### Correlation of *NUP155* expression with DNA methylation-based stem score (DNAss) and RNA methylation-based stem score (RNAss)

**Fig. 5:**
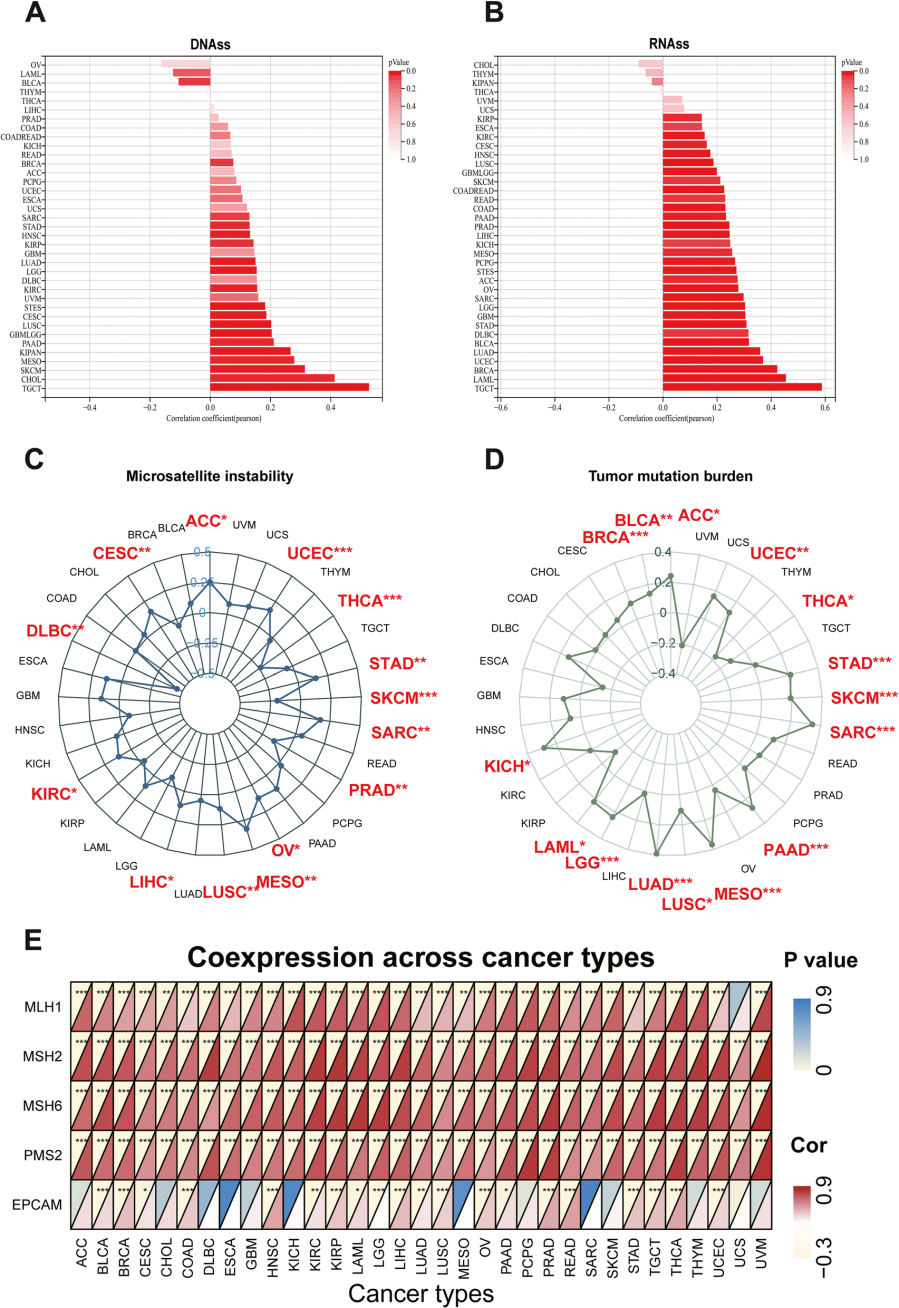
Associations between *NUP155* expression and stemness score, MSI, TMB, and MMR in pan-cancer. **A**-**B** Bar charts illustrating the relationship between *NUP155* expression and DNAss and RNAss. **C**-**D** Radar plots illustrating the relationship between *NUP155* expression and TMB as well as MSI. **E** The heat map illustrating the relationship between the expression of *NUP155* and MMR genes

The upregulation of stem cell marker expression in tumor cells is strongly correlated with tumor recurrence, metastasis, and drug resistance. The expression of *NUP155* exhibited varying degrees of correlation with DNAss **(**Fig. 5A**)** and RNAss **(**Fig. 5B**)** in different cancer types. *NUP155* expression was associated with DNAss in 19 tumors. In particular, *NUP155* expression was positively correlated with DNAss in BRCA, CESC, CHOL, glioma (GBMLGG), HNSC, KIRC, KIRP, pan-kidney cohort (KICH + KIRC + KIRP) (KIPAN), LGG, LUAD, LUSC, MESO, PAAD, stomach and esophageal carcinoma (STES), SARC, SKCM, STAD, and TGCT and negatively correlated with DNAss in BLCA. Additionally, *NUP155* expression was positively correlated with RNAss in 30 tumors. Thus, *NUP155* expression was correlated with DNAss and RNAss in several tumors and may potentially promote the activation of tumor stem cells and facilitate tumor recurrence and proliferation.

### Correlation of *NUP155* expression with TMB, MSI, and MMR genes

Immunotherapy markers are useful for screening patients who may benefit from the treatment as some patients do not respond to immunotherapy and experience severe immune-related side effects. Several clinical studies have demonstrated the promising predictive value of TMB. Tumor cells with a high TMB are easily recognized by the immune system. Consequently, immunotherapy increases the response rates and the survival rates in patients with a high TMB [38]. *NUP155* expression was positively correlated with TMB in ACC, BLCA, BRCA, KICH, LAML, LGG, LUAD, LUSC, MESO, PAAD, SARC, SKCM, STAD, and UCEC and negatively correlated with TMB in THCA **(**Fig. 5D**)**. MSI, which is characterized by deficiencies in the MMR proteins, is a well-recognized biomarker for ICI response. *NUP155* expression was positively correlated with MSI in ACC, CESC, KIRC, LIHC, LUSC, MESO, OV, SARC, STAD, and UCEC and negatively correlated with MSI in DLBC, PRAD, SKCM, and THCA **(**Fig. 5C**)**. We further explored the relationship between *NUP155* expression and MMR genes (namely MLH1, MSH2, MSH6, PMS2, EPCAM). As shown in Fig. 5E, *NUP155* expression was correlated with MMR genes in almost all cancers. These results indicate that *NUP155* expression may determine the outcomes of ICI therapy in patients with cancer by influencing TMB, MSI, and MMR.

### Correlation between *NUP155* and TIME

Previous studies have demonstrated that the complexity and diversity of TIME regulate tumorigenesis and tumor progression. Thus, this study examined the correlation between *NUP155* expression and TIME in pan-cancer. The eight tumors with the highest correlation coefficients are shown in Supplementary Fig. 5. Among these eight cancers, *NUP155* expression was negatively correlated with both stromal and immune scores in GBM, STES, STAD, and SKCM. Meanwhile, *NUP155* expression was negatively correlated with immune scores in TGCT, SARC, and KIPAN. These findings suggested a close correlation between *NUP155* expression and the tumor microenvironment in different types of cancer.

### Correlation of *NUP155* expression with tumor-infiltrating immune cells (TIICs) and immune modulator genes

**Fig. 6:**
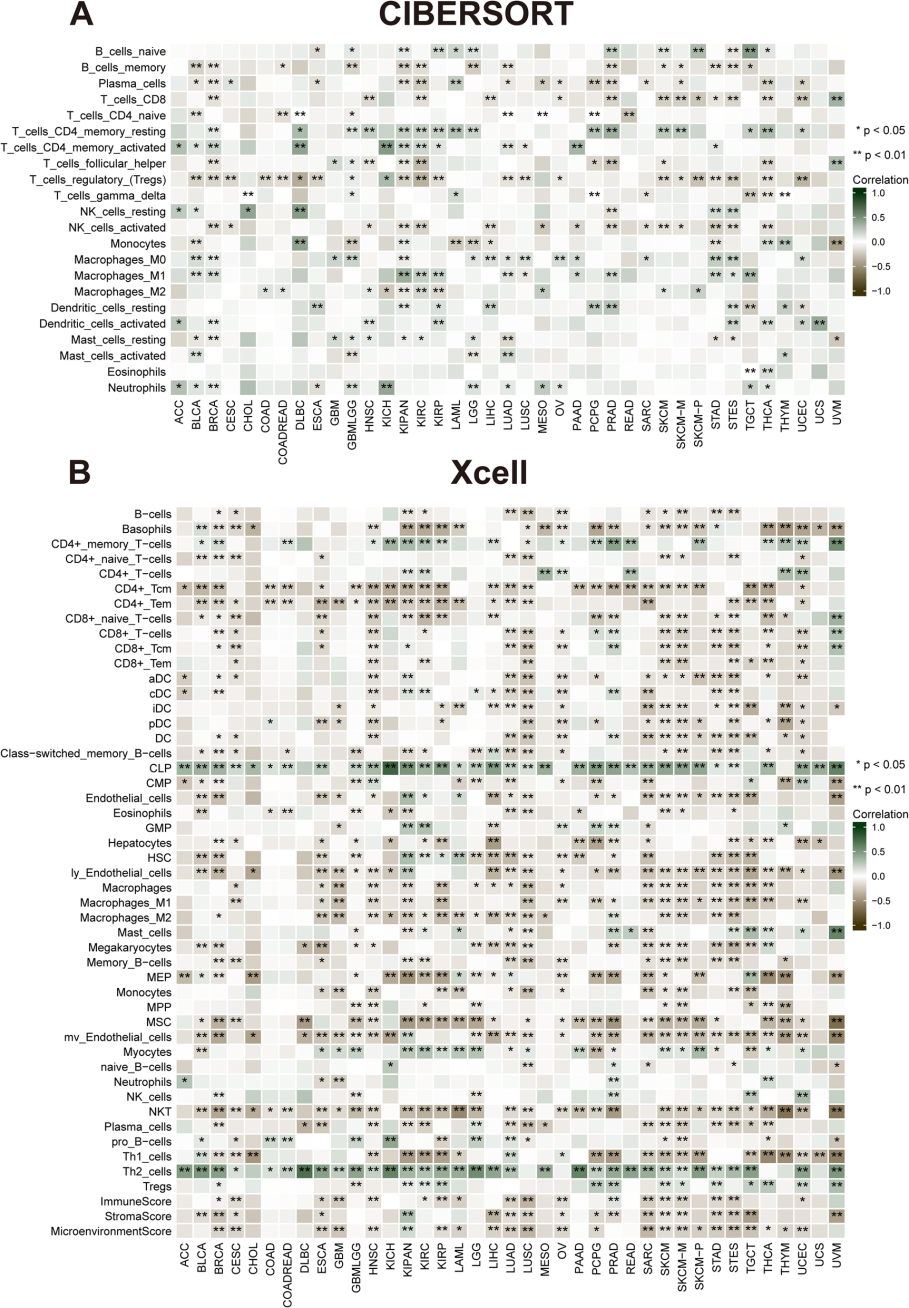
Correlation of *NUP155* expression with immune infiltration. **A** The heat map showing that *NUP155* expression correlates significantly with tumor infiltration of different immune cells from the CIBERSORT database. **B** The heat map showing that *NUP155* expression correlates significantly with tumor infiltration of different immune cells based on the xCell database

Comprehensive analysis of the correlation between *NUP155* expression and the degree of immune cell infiltration in various cancer types was performed using the xCell database. *NUP155* expression was negatively correlated with the levels of infiltrating immune cells, except CD4 + memory T cells, CD4 + T cells, common lymphoid precursors, granulocyte/macrophage precursors, myocytes, and Th2 cells **(**Fig. 6B**)**. Moreover, the levels of 26 immune cell types were examined using the “CIBERSORT” algorithm. Correlation analysis revealed that *NUP155* expression was positively correlated with the levels of infiltrating naïve B cells, CD4 + memory resting T cells, CD4 + memory activated T cells, dendritic cells, mast cells, macrophages, NK cells (resting), and neutrophils. In contrast, the levels of memory B cells, CD4 + naïve T cells, CD8 + T cells, follicular helper T cells, plasma cells, Treg cells, and activated NK cells were negatively correlated with *NUP155* expression **(**Fig. 6A**)**. Additionally, analysis at the single-cell level revealed the expression of *NUP155* in various immune cells, including CD4 + T cells, CD8 + T cells, B cells, natural killer (NK) cells, monocytes, dendritic cells, and T regulatory (Treg) cells. In particular, *NUP155* expression was upregulated in immune cells, especially in proliferative T cells (T prolif cells), Treg cells, and CD8 + exhausted T (Tex) cells, of patients with CRC, LIHC, SKCM, and NSCLC (Supplementary Fig. 6).

**Fig. 7:**
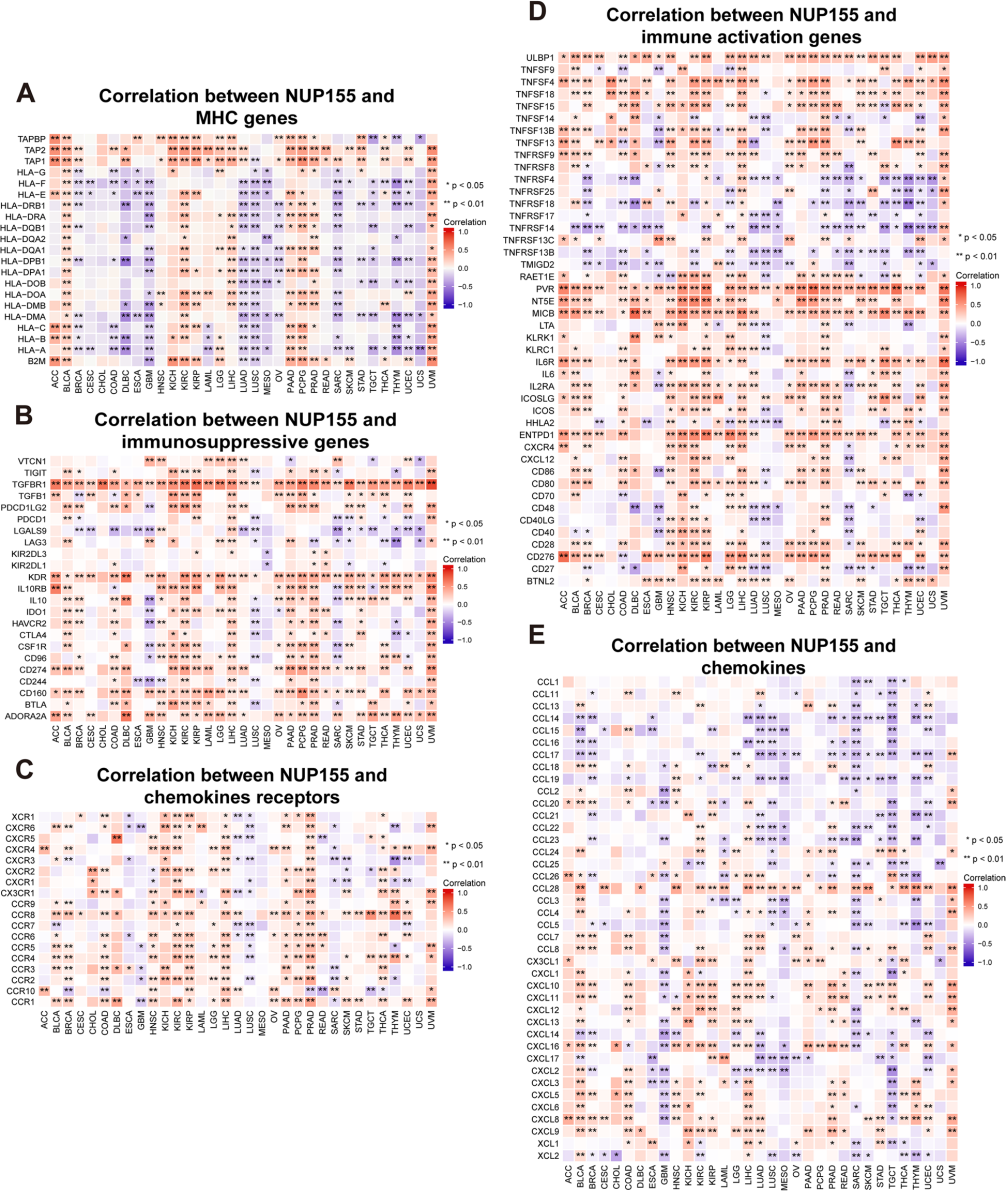
Co-expression of *NUP155* and immune-related genes in pan-cancer. **A**-**E** The heatmap represents the correlation between *NUP155* expression and MHC genes, immunosuppressive genes, chemokine receptors, immune activation genes and chemokines

Tumor-induced immunosuppression is the primary mechanism through which cancers evade immune surveillance and attack. Tumors manipulate the immune response by modulating the immune checkpoint (ICP) pathway. In this study, gene co-expression analysis was performed to investigate the correlation between *NUP155* expression and immune-related genes in various cancers. The heatmaps of the analyzed genes, including those encoding major histocompatibility complex (MHC) **(**Fig. 7A**)**, immunosuppressive factors **(**Fig. 7B**)**, chemokine receptors **(**Fig. 7C**)**, immune activation factors **(**Fig. 7D**)**, and chemokines **(**Fig. 7E**)**, revealed a strong co-expression pattern between *NUP155* and immune-related genes. *NUP155* expression was positively correlated with the expression of immune-related genes in ACC, BLCA, HNSC, KICH, KIRC, KIRP, LIHC, PAAD, PCPG, PRAD, and UVM. However, a limited number of immune-related genes exhibited co-expression with *NUP155* in CHOL.

### PPI network of *NUP155* and effect of *NUP155 *on drug response

**Fig. 8:**
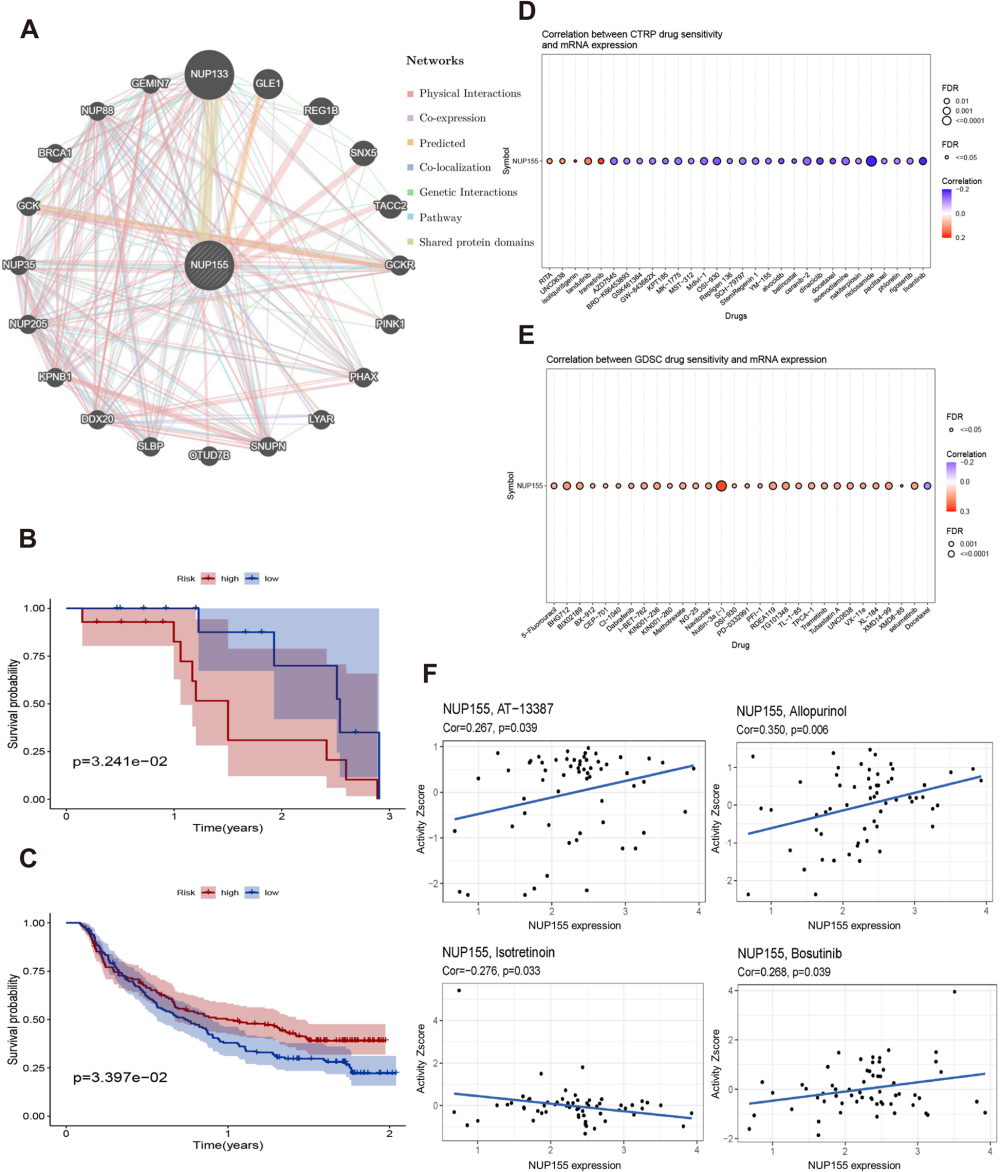
**A** a PPI network for *NUP155*. **B**-**C** Kaplan-Meier analysis of the association between *NUP155* expression and OS in the GSE78220 and Imvigor210 immunotherapy cohorts. **D**-**F** Correlation of *NUP155* expression with drug sensitivity in CTRP, GDSC and Cell Miner databases

A PPI network of *NUP155* was constructed using the GeneMANIA online program to investigate the potential role of *NUP155* in carcinogenesis. As shown in Fig. 8A and Supplementary Table S7, *NUP155* physically interacted with *NUP133*, *GLE1*, *REG1B*, *SNX5*, and *TACC2*. Next, the correlation between *NUP155* expression levels and drug sensitivity was analyzed using the CTRP and GDSC databases. In the CTRP dataset, *NUP155* expression was negatively correlated with the sensitivity to drugs, such as trametinib, tivantinib, dinaciclib, and docetaxel **(**Fig. 8D and Supplementary Table S4**)**. Meanwhile, in the GDSC dataset, *NUP155* expression was positively correlated with the sensitivity to drugs, such as nutlin-3a (-) and 5-Fu **(**Fig. 8E and Supplementary Table S5). To further investigate the correlation between *NUP155* expression and drug sensitivity in various cancer cell lines, the Cell Miner database was used. As shown in Fig. 8F, *NUP155* expression was positively correlated with sensitivity to AT-13,387, allopurinol, and bosutinib and negatively correlated with sensitivity to isotretinoin.

Additionally, the correlation between *NUP155* expression and patient prognosis after PD-1/PD-L1 immunotherapy was examined by analyzing two immunotherapy cohort datasets (GSE78220 and Imvigor210). GSE78220 comprises the data of patients with malignant melanoma who received anti-PD-1 immunotherapy, while Imvigor210 comprises the data of patients with urothelial carcinoma who received anti-PD-L1 therapy. The KM survival curve of the GSE78220 cohort revealed that *NUP155* upregulation was associated with poor OS in patients with malignant melanoma **(**Fig. 8B**)**. Meanwhile, the KM survival curve of the Imvigor210 cohort revealed that *NUP155* upregulation was associated with beneficial OS in patients with urothelial carcinoma **(**Fig. 8C**)**.

### GSEA

**Fig. 9:**
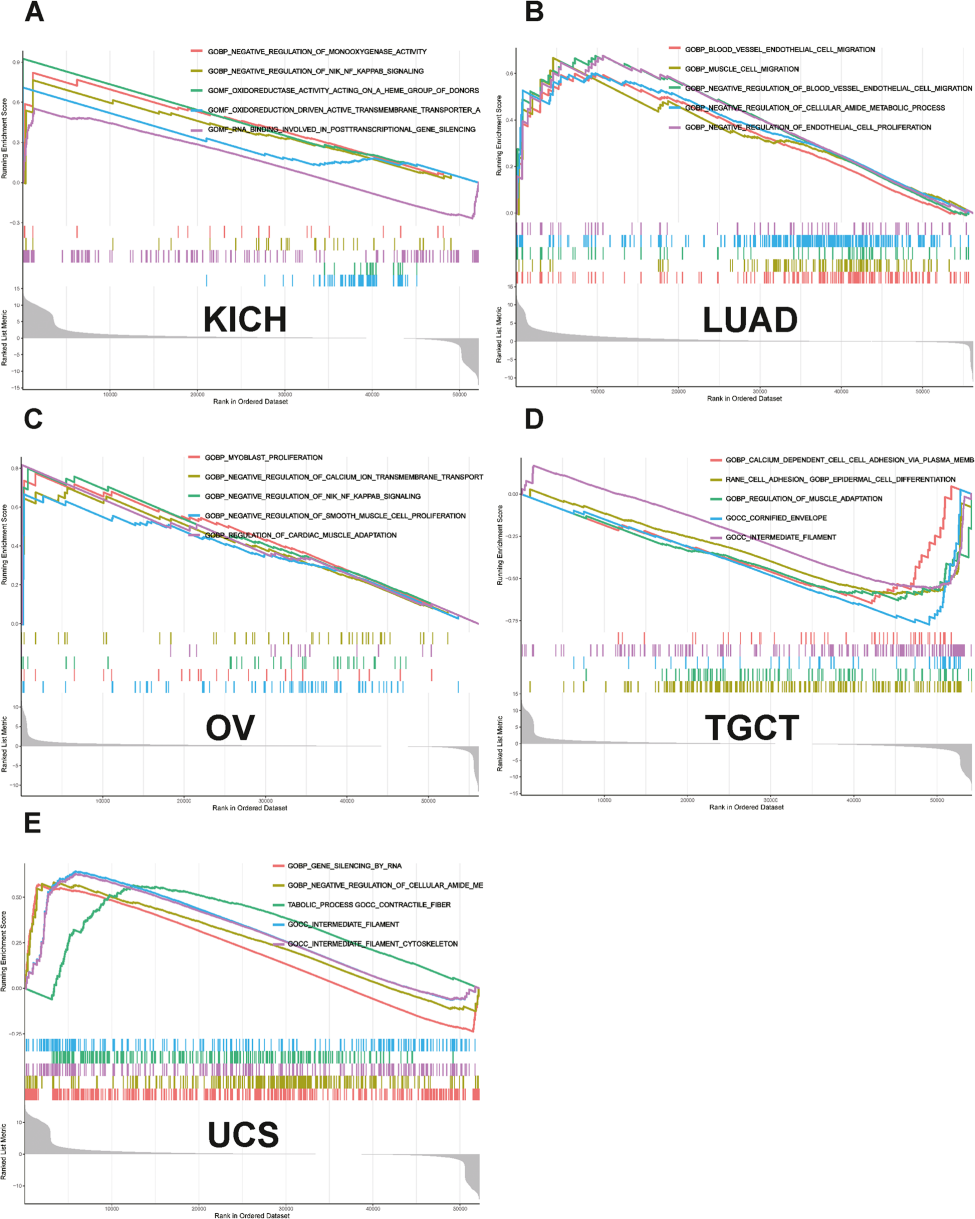
Results of GSEA. GO functional annotation of *NUP155* in various cancers, including (**A**) KICH, (**B**) LUAD, (**C**) OV, (**D**) TGCT, (**E**) UCS

**Fig. 10:**
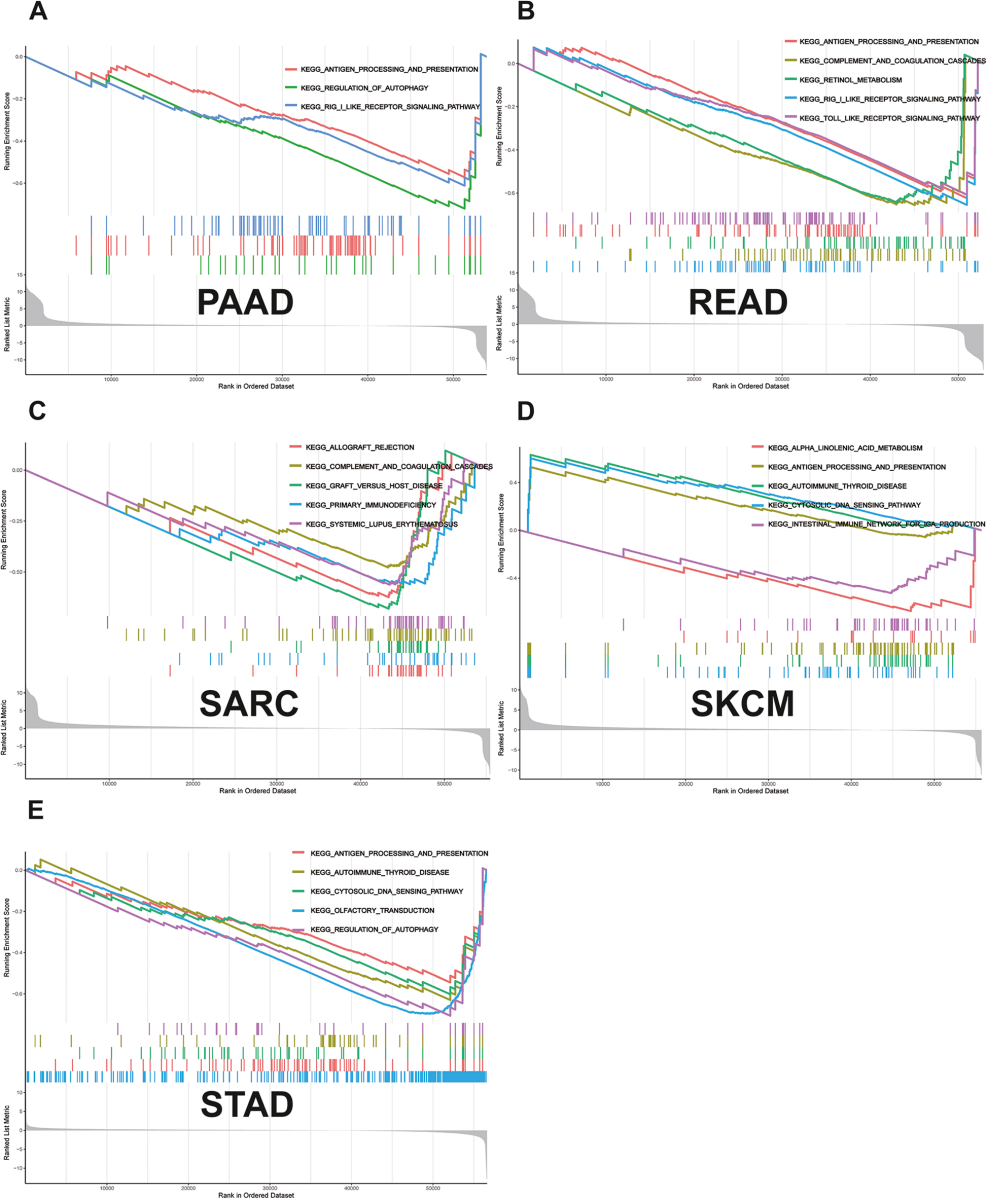
Results of GSEA. KEGG pathway analysis of *NUP155* in various cancers,  including (**A**) PAAD, (**B**) READ, (**C**) SARC, (**D**) SKCM, (**E**) STAD

GSEA revealed that *NUP155* was enriched in multiple GO terms, including the negative regulation of NIK/NF-κB signaling, intermediate filaments, and RNA-mediated gene silencing. (Fig. 9A-E) KEGG analysis indicated that *NUP155* was enriched in immune-related pathways, such as antigen processing and presentation, toll-like receptor signaling pathway, RIG-I-Like receptor signaling pathway, and allograft rejection. (Fig. 10A-E) GSEA of the REACTOME gene set collection suggested the enrichment of several immune and inflammatory functional pathways, including the class I MHC-mediated antigen processing and presentation pathway, adaptive immune system pathway, interleukin-1 signaling pathway, antigen processing via ubiquitination and proteasome degradation pathway, and MHC Class II antigen presentation pathway, in various cancers. *NUP155* was enriched in cell cycle, mitotic spindle checkpoint, regulation of *TP53* activity, DNA repair, and other pathways (Supplementary Fig. 7 and Supplementary Table S6). These findings suggest that *NUP155* has a crucial role in the inflammatory response and TIME.

### Differential expression of *NUP155* in breast cancer cells and normal breast cells

According to Cancer Statistics 2022, breast, lung, and colorectal cancers account for 51% of all newly diagnosed cases in women. In particular, breast cancer accounts for approximately one-third of cases. Therefore, the differential expression of *NUP155* between healthy breast cells (MCF-10 A cells) and three breast cancer cell lines (BT-549, MDA-MB-231, and T-47D cells) was examined using qRT-PCR analysis (Supplementary Fig. 8). The results of qRT-PCR analysis were consistent with those of bioinformatics analysis. The expression of *NUP155* mRNA in breast cancer cell lines was significantly higher than that in healthy breast cells. Triple-negative breast cancer (TNBC) has the worst prognosis and poses significant treatment challenges among breast cancer subtypes, with a 5-year survival rate of only 11% in advanced stages [39]. Two TNBC cell lines (MDA-MB-231 and BT-549 cells) were used in subsequent in vitro experiments.

### Effect of *NUP155* on the proliferation, migration, and apoptosis of TNBC cells

**Fig. 11:**
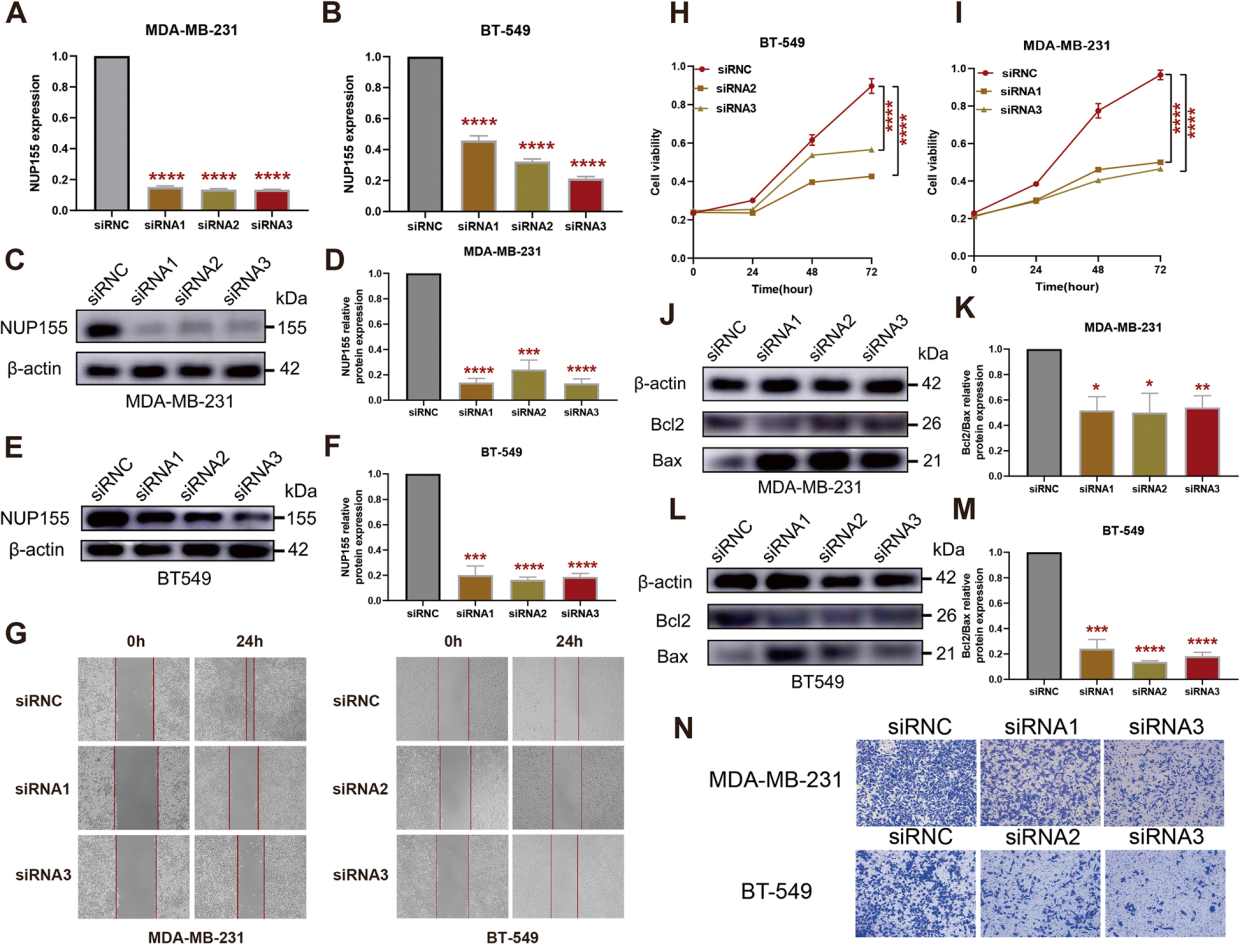
Effect of *NUP155* silencing on TNBC cell lines MDA-MB-231 and BT-549. **A**-**B** RT-PCR validation of *NUP155* silencing efficiency. Western blot analysis to verify *NUP155* silencing efficiency in MDA-MB-231 cells (**C**-**D**) and BT-549 cells (**E**-**F**). **G** Wound healing assay to analyze the impact of *NUP155* silencing on TNBC cell healing ability. **H**-**I** CCK8 assay to analyze the effects of *NUP155* silencing on the proliferation of MDA-MB-231 and BT-549 cells. Western blot analysis of the decrease in the BCL2/BAX expression ratio in MDA-MB-231 cells (**J**-**K**) and BT-549 cells (**L**-**M**) due to *NUP155* siRNA. **N** Transwell assay to analyze the impact of *NUP155* silencing on cell migration. *p < 0.05; **p < 0.01; ***p < 0.001; ****p < 0.0001. The blots were cut prior to hybridisation with antibodies during blotting, and the three replicates of original blots of Fig. 11C, E and J, and Fig. 11L are presented in the Supplementary material

To investigate the effect of *NUP155* on TNBC, si-*NUP155* was transfected into MDA-MB-231 and BT-549 cells. Transfection with si-*NUP155* downregulated the mRNA and protein expression levels of NUP155 **(**Fig. 11A–F**)**. Western blotting analysis revealed that the BCL2/BAX expression ratio was significantly downregulated in si-NUP155-transfected TNBC cells **(**Fig. 11J–M**)**. The CCK-8 assay results revealed that transfection with si-*NUP155* significantly decreased tumor cell proliferation **(**Fig. 11H–I**)**. Furthermore, the wound healing and transwell assay results revealed that *NUP155* knockdown significantly impaired the wound healing **(**Fig. 11G**)** and migratory **(**Fig. 11N**)** abilities of TNBC cells.

## Discussion

The NPC, a giant protein complex embedded in the nuclear envelope, mediates selective nucleocytoplasmic transport [40]. Deficiency in NPC, which has a crucial role in gene expression and growth and development, is associated with the pathogenesis of various pathological conditions, such as viral infections, cancer, and neurodegenerative diseases. Thus, the nuclear transport machinery is a therapeutic target for several diseases [41]. Previous studies have reported that the NPC promotes tumorigenesis in hematological cancers and non-hematological malignancies, such as skin, lung, pancreatic, prostate, and colon cancers [42]. Among nuclear pore proteins, NUP155 is critical for assembling the structure of the NPC [43]. *NUP155* is involved in mitotic arrest mediated by the novel anti-tumor drug NP-10 [44] and regulates mRNA translation for the cell cycle protein-dependent kinase inhibitor *p21* [20]. Therapeutic approaches for cancer mainly target the proliferation of cancer cells, impairing the assembly of the mitotic spindle to arrest cancer cell division and death. This approach is considered to be the most effective therapeutic strategy. This is the reason why we focus on the gene *NUP155*. The role of *NUP155* in different cancer types has not been systematically examined using bioinformatic approaches. This study aimed to comprehensively analyze the differential expression, prognostic value, and biological function of *NUP155* in different cancer types. The correlation of *NUP155* with TIME, TIICs, and immune-related genes was also investigated.

This study demonstrated that *NUP155* was under-expressed in normal human tissues, except for bone marrow and testis. We hypothesized that *NUP155* upregulation is related to enhanced cell proliferation and turnover in the bone marrow and testis. *NUP155* is upregulated in most cancer types but is downregulated in LAML and TGCT. Bone marrow contains hematopoietic stem cells, while testis contains spermatogonial stem cells. Several studies have reported that NPC is important for maintaining stem cell homeostasis [45]. For example, the inhibition of *NUP153* can lead to the derepression of developmental genes and the induction of early differentiation in stem cells [46, 47]. Therefore, we hypothesize that *NUP155* upregulation in healthy bone marrow and testis is necessary to maintain stem cell homeostasis and that the suppression of *NUP155* expression in LAML and TGCT leads to aberrant proliferation and differentiation of stem cells. The NPC plays a major role in cell fate determination. *NUP98* mutations contributing to leukemia development have been extensively studied. Mutations in multiple nucleoporin-encoding genes can cause tissue-specific defects or lethality in animals [48–50]. Based on the data shown in Fig. 3B, we speculate that *NUP155* may also influence leukemia through gene mutations. Although the expression level of *NUP155* is downregulated in TGCT, the data in Fig. 3A revealed that the *NUP155* promoter methylation level is downregulated in TGCT, indicating gene instability.

Cancer cells are characterized by an overall loss of methylation modifications and aberrant methylation sites within the enhancer and promoter regions [51, 52]. The *NUP155* promoter methylation level is downregulated in COAD, PRAD, READ, and TGCT, which is consistent with the classical model [53]. However, the *NUP155* promoter hypermethylation upregulates *NUP155* expression in BRCA, CESC, ESCA, HNSC, KIRC, LIHC, LUAD, LUSC, PAAD, SARC, and UCEC tissues. A review by Jim Smith et al. in ‘Trends in Cancer’ suggested that promoter DNA hypermethylation promotes aberrant gene activation. The authors further discussed the potential molecular mechanisms underlying this aberrant regulation [54]. Therefore, the correlation between *NUP155* expression and DNA methylation identified in this study warrants further investigation.

Somatic mutations that accumulate in normal tissues are associated with aging and disease. Additionally, somatic mutations enable the development of novel therapeutic approaches for cancer [55]. Similarly, tumor-specific antigens derived from somatic mutations have provided new approaches for developing cancer therapy [56]. Designing vaccines based on patient-specific mutations is a potential strategy for developing personalized tumor therapy [57]. In this study, *NUP155* was frequently mutated in various tumors, especially melanoma, endometrial carcinoma, cervical adenocarcinoma, BLCA, and cervical squamous cell carcinoma. These findings demonstrated that *NUP155* is a potential target for cancer vaccines, especially for melanoma, which was the most frequent tumor type. Cox regression analysis of TCGA dataset revealed that *NUP155* upregulation is a risk factor for OS in 13 types of cancer. Additionally, *NUP155* upregulation was a risk factor for DSS and PFS in nine types of tumors and a favorable factor for DSS and PFS in KIRC. These findings suggest that *NUP155* can be used to stratify patients with cancer.

TMB is a valuable predictive biomarker for immunotherapy response in various cancer types [58]. Meanwhile, MSI is an important biomarker for ICI response [59]. The upregulation of MSI or TMB can lead to the generation of potent neoantigens, which elicit enhanced immune responses and contribute to an enhanced immunotherapeutic response [59, 60]. The findings of this study indicate a strong correlation between *NUP155* expression and the levels of TMB and MSI in various cancer types. Hence, *NUP155* expression can aid in predicting patient response to ICI therapy.

The results of this study suggest that *NUP155* plays a crucial role in cancer immunity. The ESTIMATE score revealed a negative correlation between *NUP155* expression and the levels of stromal and immune cells in the tumor microenvironment of 15 different cancer types. TIICs regulate tumorigenesis and tumor progression [61]. Under physiological conditions, the immune system can recognize and destroy tumor cells in the TIME. However, tumor cells can evade the immune system through various mechanisms that promote their survival and growth. Cytotoxic T cells expressing CD8 receptors on their surface play a pivotal role in the response to cancer immunotherapies. CD8 receptors are the most potent effectors in the anti-cancer immune response [62]. Treg cells contribute to resistance against ICI therapies, promoting cancer progression [63]. Th1 cytokines stimulate immune cells to eliminate tumor cells, while Th2 cytokines inhibit tumor immune responses [64, 65]. Analysis of immune cell infiltration using the xCell database revealed that *NUP155* expression was negatively correlated with the infiltration levels of CD8 + cells and Th1 cells and positively correlated with the infiltration levels of Treg cells and Th2 cells. T cell exhaustion refers to the impaired state of CD8 + T cells, which can identify and eliminate tumor cells, leading to a diminished response against tumor cells [66, 67] Dysfunctional CD8 + Tex cells in the tumor microenvironment exhibit the expression of immune co-inhibitory receptors, including LAG3, CD160, CTLA4, and TIGIT [68, 69]. CD8 + Tex cells with enhanced expression of ICP receptors exhibit an exhausted phenotype [70]. As shown in Fig. 7B, *NUP155* was positively correlated and co-expressed with these ICP receptors in most tumors. Hence, we hypothesized that *NUP155* may upregulate ICP receptors, regulating the levels of CD8 + Tex cells and consequently modulating the TIME. Additionally, analysis of the TISCH dataset revealed that *NUP155* was upregulated in T prolif cells and Treg cells. Analysis of the xCell dataset and the TISCH dataset revealed that *NUP155* upregulation may modulate the tumor microenvironment status by upregulating the levels of Tregs and regulating the balance of Th1 and Th2 cells. GSEA revealed that *NUP155* was significantly enriched in immune-related pathways, especially those involved in antigen processing and expression. Tumor cells can evade immune recognition by disrupting antigen processing and expression through the suppression of dendritic cell function and the downregulation of HLA-1 [71]. Immune cell infiltration analysis revealed that *NUP155* expression was negatively correlated with the infiltration levels of dendritic cells. These findings suggested that *NUP155* expression is a prognostic risk factor in most tumor types. ICIs exert potent growth-inhibitory effects against various cancers, improving the clinical outcomes of patients with cancer [72]. In this study, *NUP155* expression was correlated with genes encoding MHC, immune suppressors, immune activators, chemokines, and chemokine receptors. In particular, *NUP155* was negatively correlated with genes encoding ICPs. Thus, *NUP155* may mediate the effects of immunotherapy in patients with cancer by regulating TIICs and ICPs.

In the PPI network of *NUP155*, the top five genes that were most strongly correlated with *NUP155* were *NUP133*, *GLE1*, *REG1B*, *SNX5*, and *TACC2*. The structure of NUP133, a nucleoporin, is similar to that of NUP155 [73, 74]. NUP133 functions as a gene regulator and promotes the expression of the oncogene *MYC* [75]. The amino (N)-terminal region of GLE1 interacts with NUP155 [76]. GLE1, an RNA export protein, is crucial for multiple steps in gene expression, from mRNA export to translation [77]. Mutations in *GLE1* can lead to developmental and neurodegenerative disorders and some cancers [78–80]. *REG1B*, *SNX5*, and *TACC2* are reported to be oncogenes [81–85]. Additionally, the PPI network revealed that *NUP155* was mainly related to functions, such as nuclear transport, nucleocytoplasmic transport, regulation of ATP metabolic process, and RNA transport as shown in Supplementary Table S8. Therefore, aberrant *NUP155* expression may interfere with these functions and activate oncogenes, such as *REG1B*, *SNX5*, and *TACC2* to exert carcinogenic effects.

*NUP155* can also serve as a predictive biomarker of immunotherapeutic response in some cancers. ICI therapy is associated with survival benefits in patients with upregulated ICP expression. PD-1(PDCD1) and PD-L1(CD274) are the most widely recognized prognostic predictors of immunotherapy [86, 87]. KM survival analysis of the immunotherapy cohort revealed that the prognosis of patients with SKCM exhibiting *NUP155* upregulation was poor, which may be related to the correlation between *NUP155*, ICP-encoding genes, and the degree of immune cell infiltration. In SKCM, PD-1 expression and dendritic cell levels were negatively correlated with *NUP155* expression. Thus, the group exhibiting *NUP155* upregulation may not benefit from PD-1 inhibitor therapy. In BLCA, the survival benefit of immunotherapy was significant in the group with *NUP155* upregulation. PD-1/PD-L1 and dendritic cells were positively correlated with *NUP155* expression in BLCA. Hence, we hypothesized that the correlation between *NUP155* expression, ICP-encoding gene expression, and the degree of immune cell infiltration affects the response of patients with cancer to immunotherapy.

The establishment of the sensitivity of tumors with differential expression levels of *NUP155* to anti-tumor drugs may guide tumor treatment. For example, trametinib, a representative MEK inhibitor, is used as a monotherapy for unresectable or metastatic melanoma with BRAF-V600E or V600K mutations [88, 89]. The sensitivity to trametinib is significantly and positively correlated with the expression of NUP155. Therefore, patients with drug-resistant melanoma exhibiting *NUP155* upregulation may be suitable for treatment with trametinib. Paclitaxel and 5-fluorouracil (5-FU) are also common chemotherapy drugs [90, 91]. *NUP155* is negatively correlated with the sensitivity to paclitaxel and positively correlated with the sensitivity to 5-FU. Therefore, tumors with *NUP155* upregulation may be resistant to paclitaxel but not to 5-FU. Analysis of *NUP155* expression can aid in selecting anti-tumor drugs in clinical practice, especially for drug-resistant tumors.

The role of *NUP155* in BRCA was validated using molecular biology methods. qRT-PCR analysis revealed that the *NUP155* mRNA level was upregulated in BRCA cells. TNBC, which accounts for 10–20% of all diagnosed breast cancers [92], is characterized by the absence of estrogen receptor, progesterone receptor, and human epidermal growth factor receptor 2 [93, 94]. Additionally, TNBC exhibits high recurrence, metastasis, and resistance to conventional treatments. Thus, the treatment of TNBC is challenging when compared with that of other types of breast cancer [95]. Clinically, TNBC is often classified as “difficult-to-treat breast cancer” and is a research hotspot in the field of breast cancer research [96, 97]. Therefore, this study selected TNBC cells for subsequent in vitro experiments to validate the findings of bioinformatics analysis. Cellular experiments revealed that *NUP155* knockdown significantly inhibited the proliferation and migration and promoted apoptosis in TNBC cells. These findings confirm the accuracy and reliability of the pan-cancer bioinformatics analysis in BRCA. The specific pathogenic mechanism of *NUP155* in breast cancer will be validated in the future.

This study has some limitations. Although *NUP155* expression was demonstrated to be associated with the immune microenvironment and prognosis of human malignancies, the regulatory effect of *NUP155* on the clinical survival rates mediated through the immune-related pathway is unclear. Additionally, this study performed preliminary experiments on BRCA but did not examine the molecular mechanisms of *NUP155* in BRCA. This systematic pan-cancer analysis suggested that *NUP155* was differentially expressed between non-cancerous and cancer tissues and that *NUP155* dysregulation is associated with tumor staging and can be used to predict the prognosis. Additionally, DNA methylation, TMB, MSI, cancer stemness, TIME, and immune cell infiltration may be correlated with *NUP155* dysregulation in cancer. These findings can aid in determining the role of *NUP155* in tumor development and progression and facilitate the application of precise and personalized immunotherapies.

### Supplementary Information

**Additional file 1:**  The original blots of western blotting.

**Additional file 2:** **Supplementary figure 1.** The correlation between *NUP155* expression and the pathological or clinical stages of cancers, including (A) ACC, (B) KICH, (C) KIRC, (D) KIRP, (E) LIHC, (F) OV, (G) SKCM, (H) UCS.

**Additional file 3:** **Supplementary figure 2.** (A) Association between *NUP155* CNV and mRNA in pan-cancer. (B) The top six with the highest correlation scores between *NUP155* CNV and mRNA.

**Additional file 4:** **Supplementary figure 3.** Association between *NUP155* expression levels and Disease-free survival (DSS) in TCGA pan-cancer. (A) Forest plot of association of *NUP155* expression and DSS. (B–J) Kaplan-Meier analysis of the association between *NUP155* expression and DSS.

**Additional file 5:** **Supplementary figure 4.** Association between *NUP155* expression levels and Progression-free survival (PFS) in TCGA pan-cancer. (A) Forest plot of association of *NUP155* expression and PFS. (B–K) Kaplan-Meier analysis of the association between *NUP155* expression and PFS.

**Additional file 6:** **Supplementary figure 5.** Eight tumors with the highest correlation coefficients between *NUP155* expression and the tumor microenvironment. (A) Correlation between *NUP155* and stromal scores in GBM, STES, TGCT, SKCM, SARC, KIPAN, STAD, KIRC. (B) Correlation between NUP155 and immune scores in GBM, STES, UCEC, THYM, ESCA, STAD, LUSC, SKCM.

**Additional file 7:** **Supplementary figure 6.** Association between *NUP155* gene and the TIME in pan-cancer tissues, using the TISCH database.

**Additional file 8:** **Supplementary figure 7.** Reactome functional annotation of *NUP155* in various cancers, including (A) CHOL, (B) PCPG, (C) KICH, (D) TGCT, (E) KIRC, (F) THCA, (G) PAAD, (H) THYM.

**Additional file 9:** **Supplementary figure 8.** Relative mRNA expression of *NUP155* in normal breast cell and breast cancer cell lines. *p < 0.05 and **p < 0.01.

**Additional file 10:** **Supplementary table S1.** The R-Scripts and online tools in this study. **Supplementary table S2.** The detail results of the methylation levels of *NUP155* in pan-cancer. **Supplementary table S3.** The landscape of *NUP155* copy number variation (CNV) in different cancer types. **Supplementary table S4.** The correlation between *NUP155* expression levels and drug sensitivity in the CTRP database. **Supplementary table S5.** The correlation between *NUP155* expression levels and drug sensitivity in the GDSC database. **Supplementary table S6.** The enrichment analysis of *NUP155* in the REACTOME pathway. **Supplementary table S7.** Genes interacting with *NUP155* in the PPI network. **Supplementary table S8.** Functional enrichment of genes interacting with *NUP155* in the PPI network.

## Data Availability

Publicly available database analyzed in this study can be found in the TCGA (https://tcgadata.nci.nih.gov/tcga/) UCSC (https://xenabrowser.net/datapages/), CellMiner (https://discover.nci.nih.gov/cellminer/home.do) and GSEA (https://www.gsea-msigdb.org/gsea/downloads.jsp).
