# Supplementary figures and images for: Pan-cancer analysis of NUP155 and validation of its role in breast cancer cell proliferation, migration, and apoptosis
Source: BMC Cancer. 2024 Mar 19;24:353. doi: 10.1186/s12885-024-12039-6 (PMC10953186; doi:10.1186/s12885-024-12039-6)

Figure 11C

NUP155  
155Kd

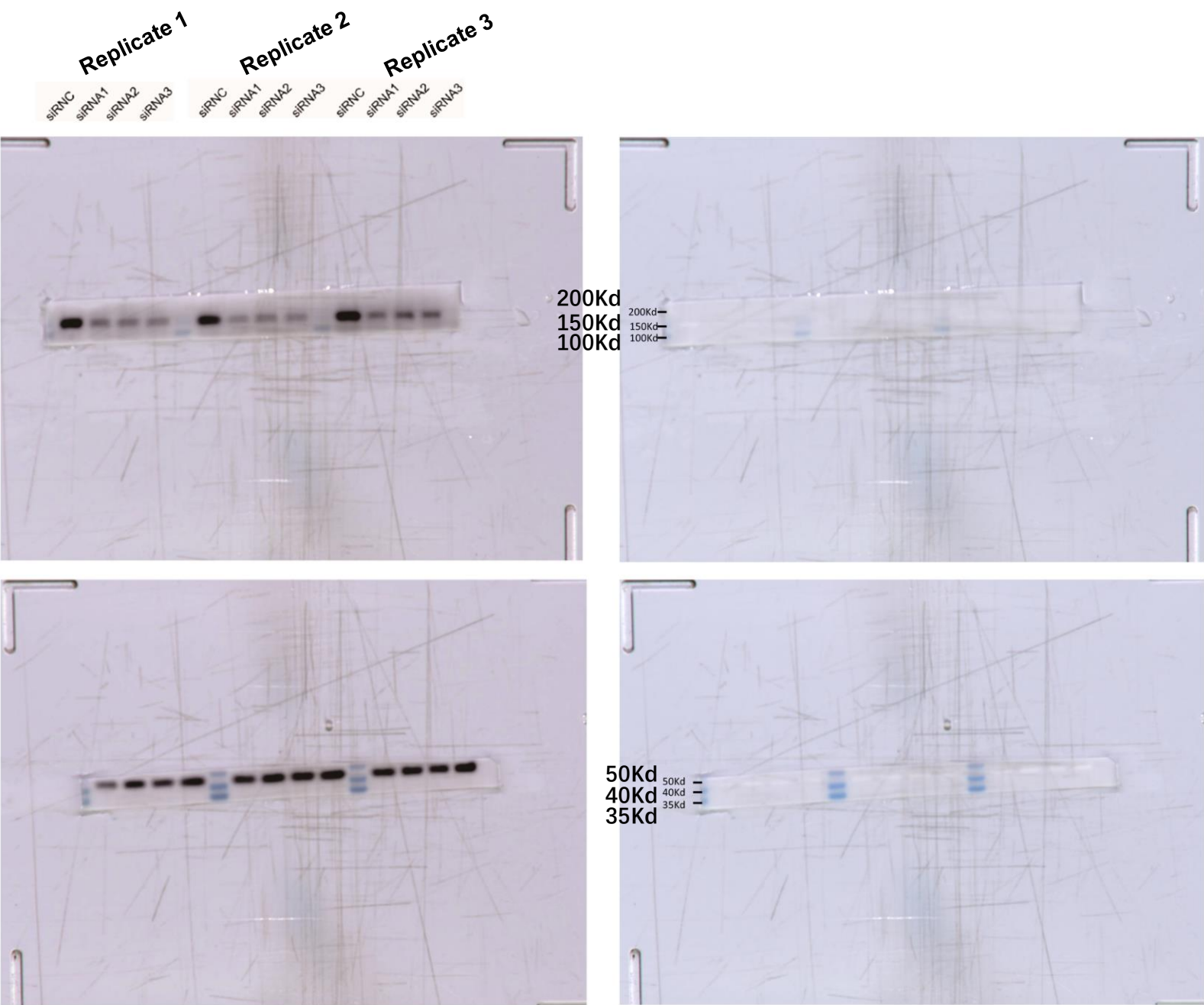

$\beta$ -actin  
42Kd

Figure 11E

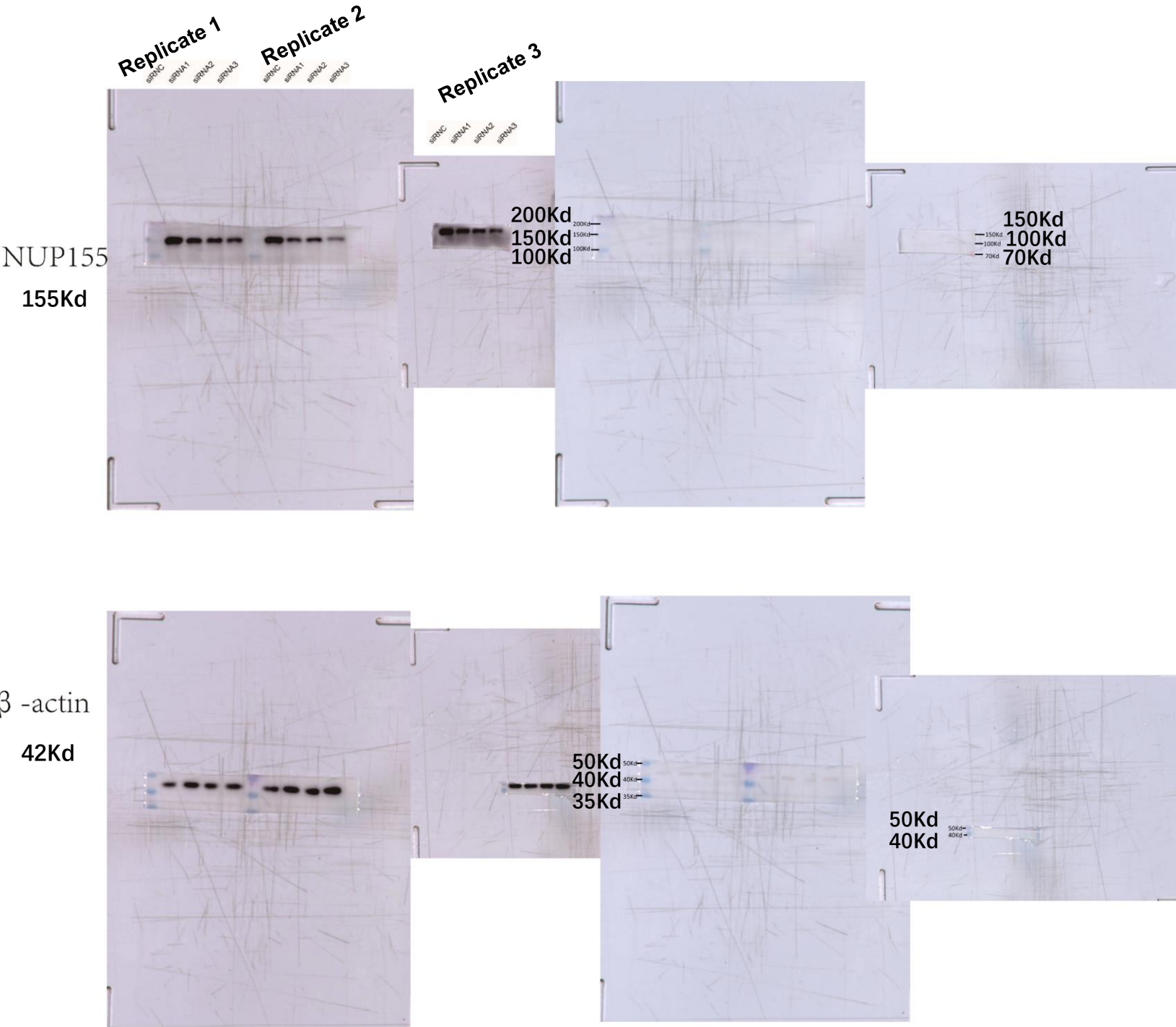

Figure 11J

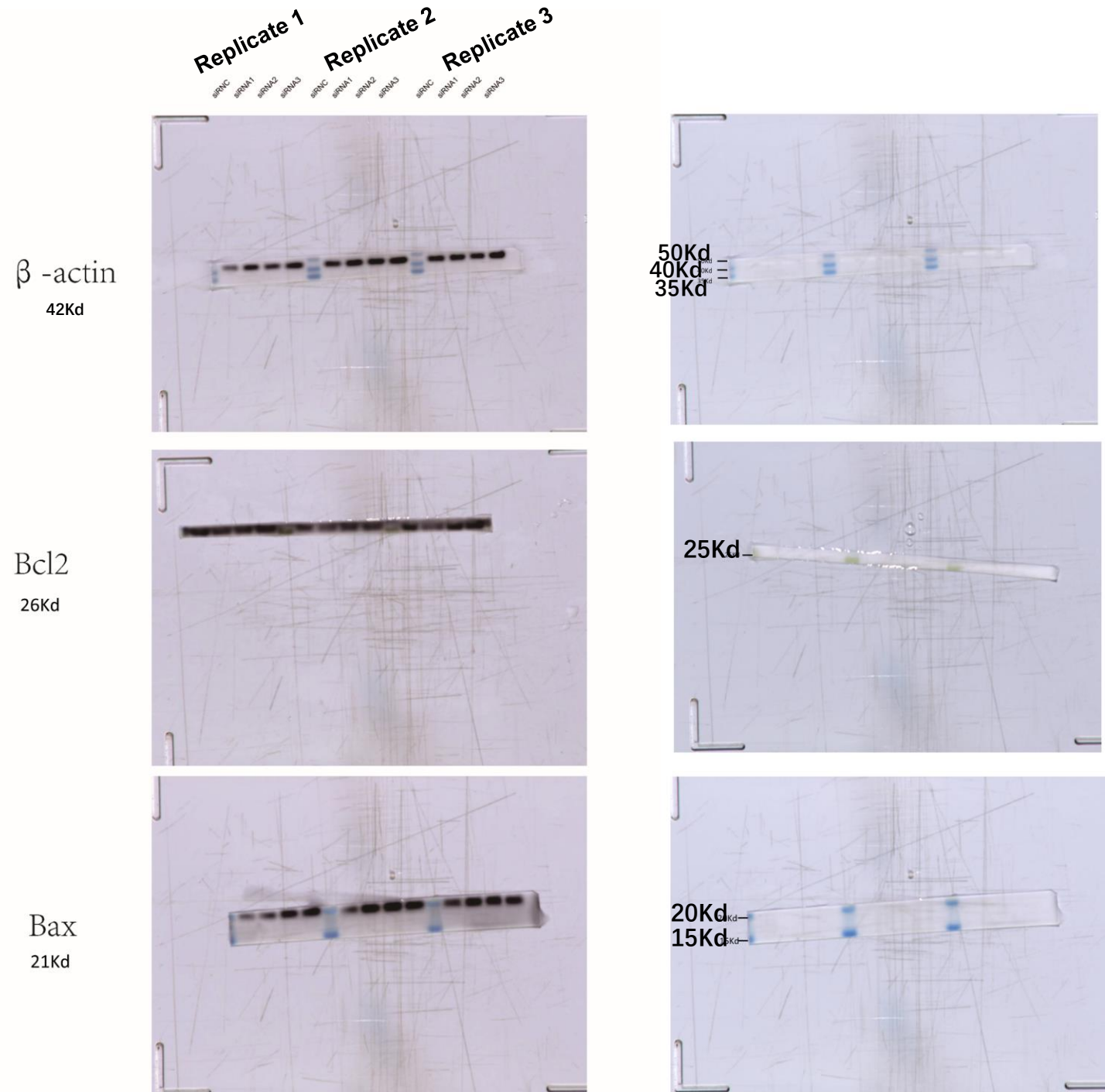

Figure 11L

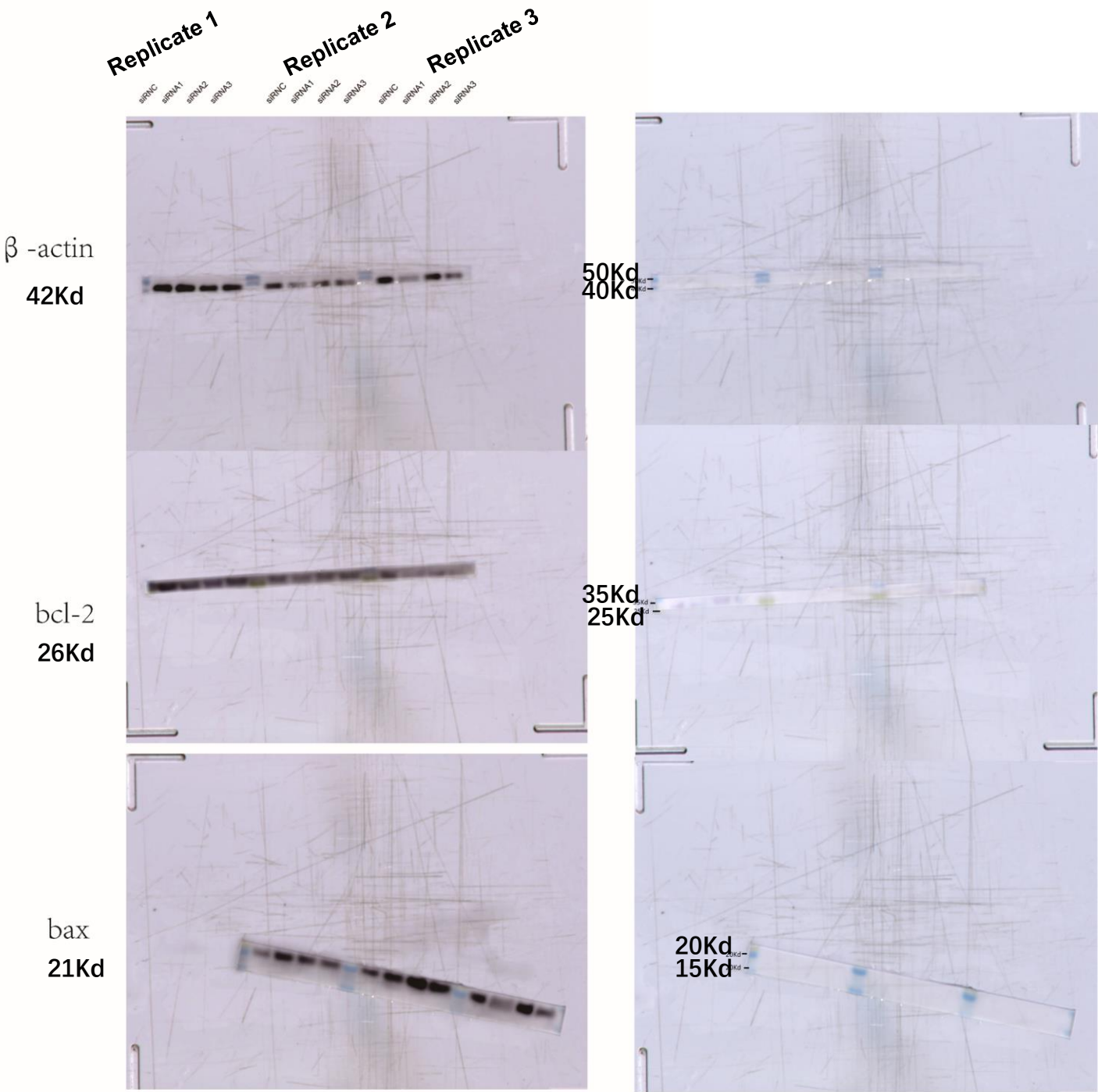

Supplement: Supplementary file 1 — Additional file 1: The original blots of western blotting. [file 12885_2024_12039_MOESM1_ESM.pdf]
