# Supplementary material for: Pan-cancer analysis of NUP155 and validation of its role in breast cancer cell proliferation, migration, and apoptosis
Source: BMC Cancer. 2024 Mar 19;24:353. doi: 10.1186/s12885-024-12039-6 (PMC10953186; doi:10.1186/s12885-024-12039-6)
Supplement: Supplementary file 2 — Additional file 2: Supplementary figure 1. The correlation between NUP155 expression and the pathological or clinical stages of cancers, including (A) ACC, (B) KICH, (C) KIRC, (D) KIRP, (E) LIHC, (F) OV, (G) SKCM, (H) UCS. [file 12885_2024_12039_MOESM2_ESM.docx]

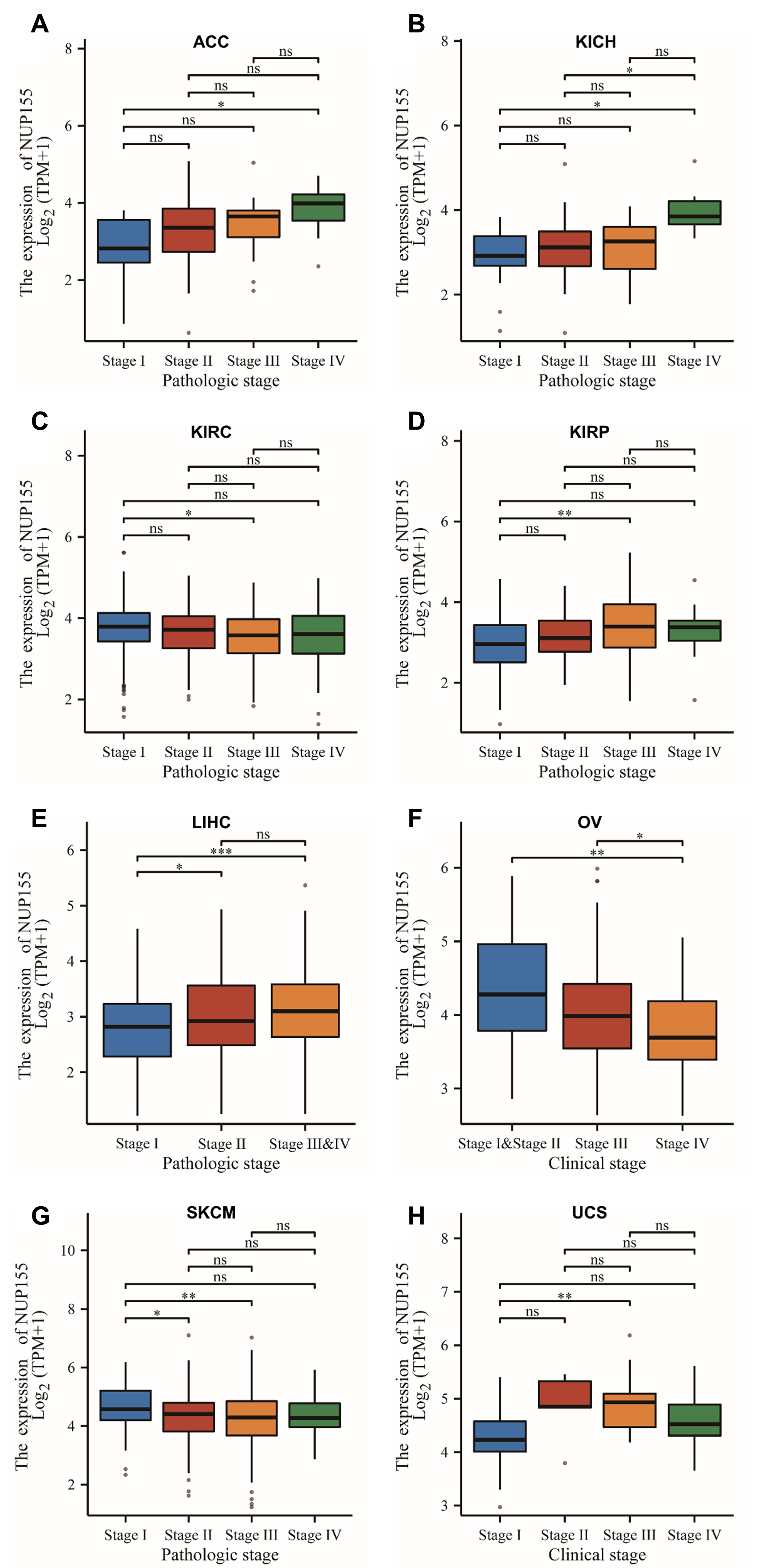


**Supplementary Figure 1:** The correlation between *NUP155* expression and the pathological stages of cancers, including (A) ACC, (B) KICH, (C) KIRC, (D) KIRP, (E) LIHC, (F) OV, (G) SKCM, (H) UCS.


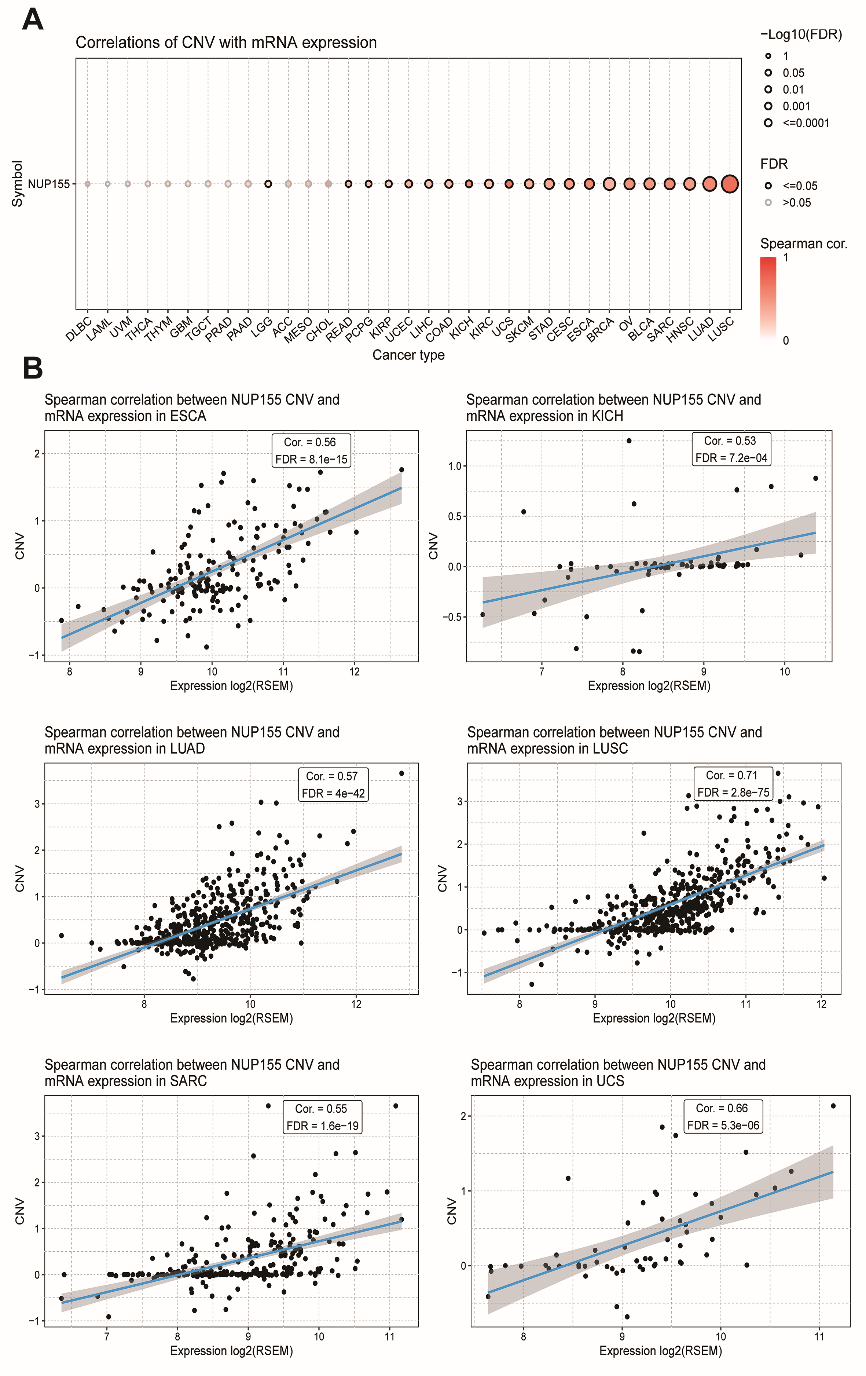


**Supplementary Figure 2:** (A) Association between *NUP155* CNV and mRNA in pan-cancer. (B) The top six with the highest correlation scores between *NUP155* CNV and mRNA.


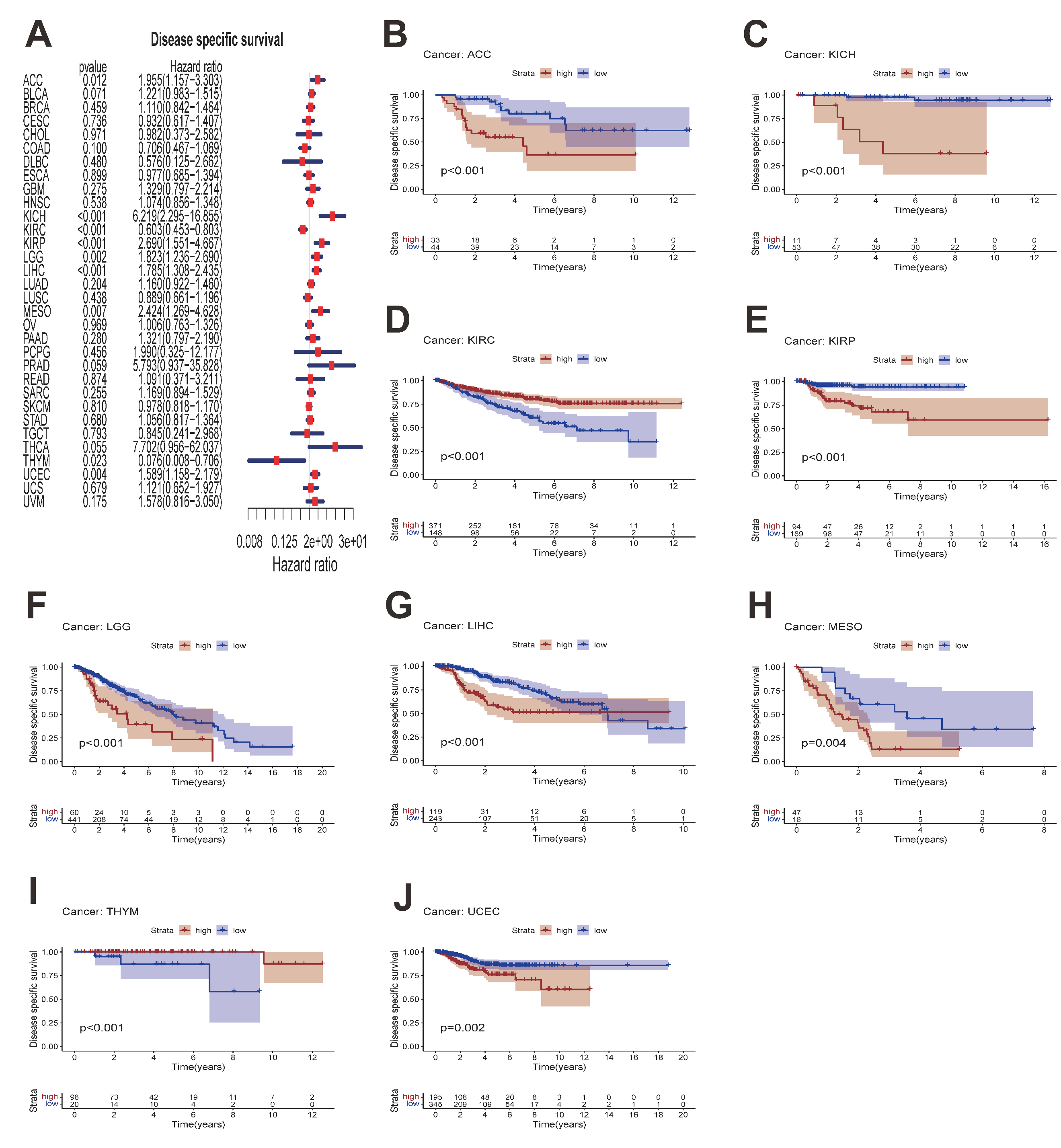


**Supplementary Figure 3:** Association between *NUP155* expression levels and Disease-free survival (DSS) in TCGA pan-cancer. (A) Forest plot of association of *NUP155* expression and DSS. (B–J) Kaplan-Meier analysis of the association between *NUP155* expression and DSS.


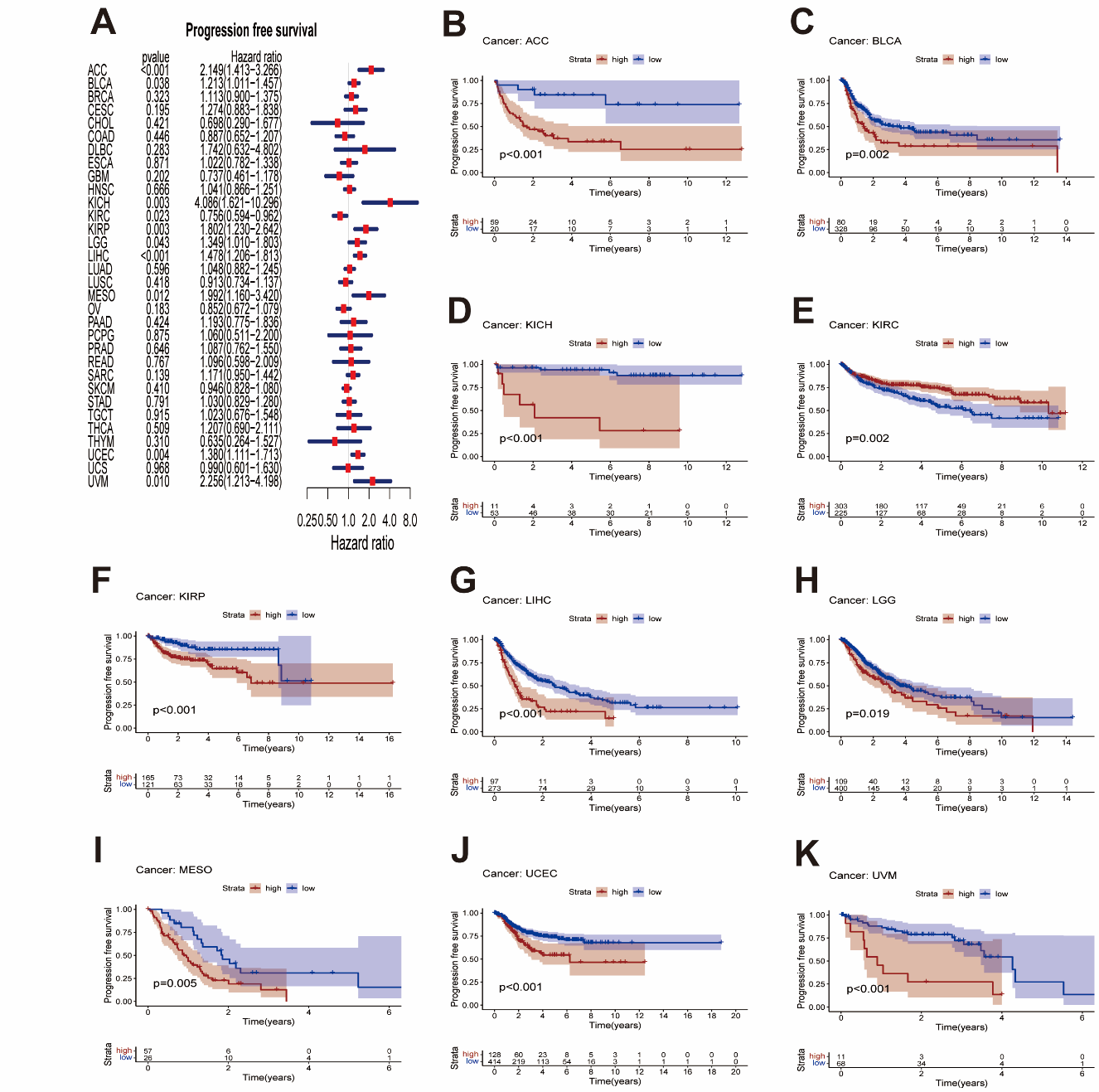


**Supplementary Figure 4:** Association between *NUP155* expression levels and Progression-free survival (PFS) in TCGA pan-cancer. (A) Forest plot of association of *NUP155* expression and PFS. (B–K) Kaplan-Meier analysis of the association between *NUP155* expression and PFS.


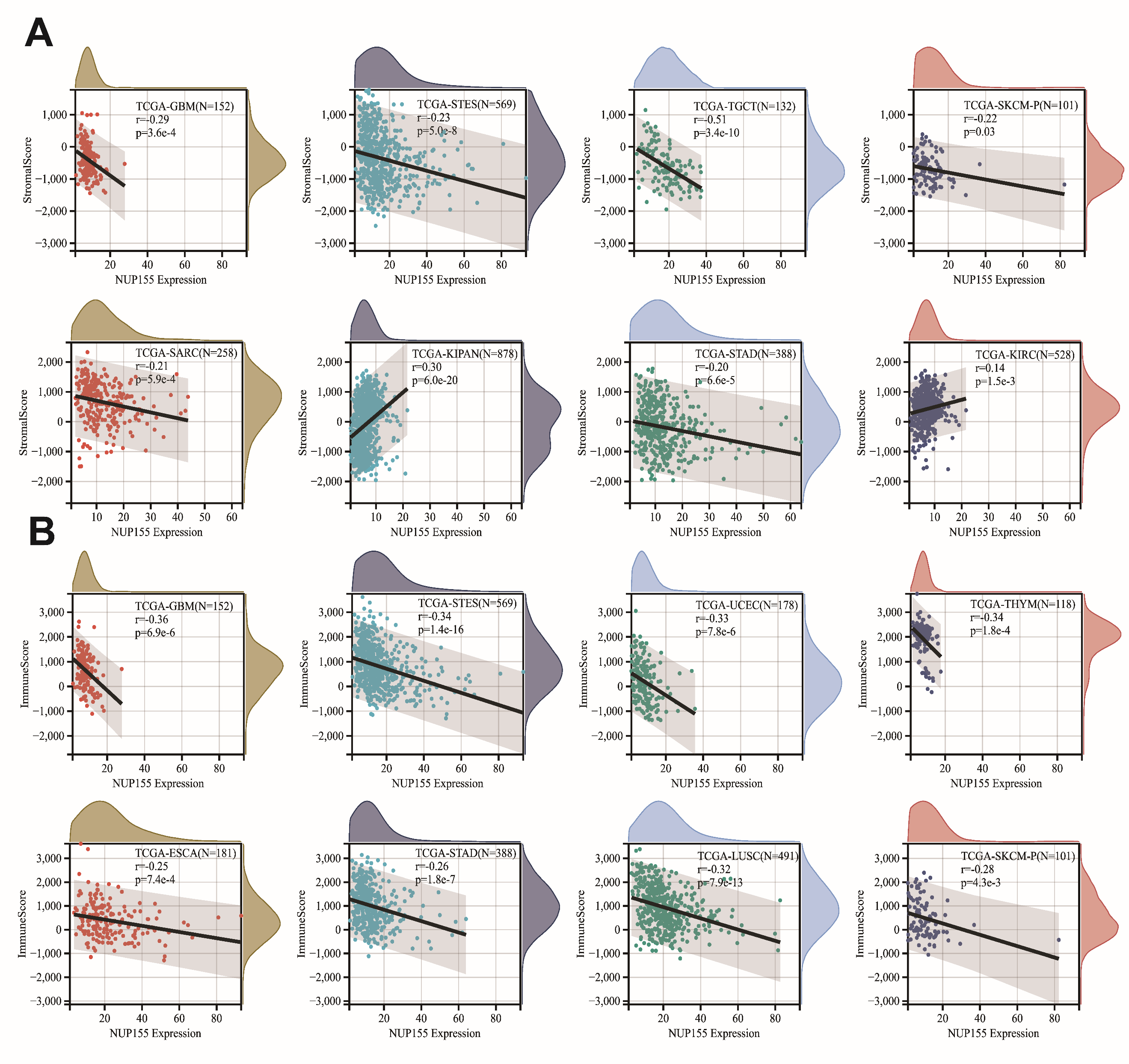


**Supplementary Figure 5:** Eight tumors with the highest correlation coefficients between *NUP155* expression and the tumor microenvironment. (A) Correlation between NUP155 and stromal scores in GBM, STES, TGCT, SKCM, SARC, KIPAN, STAD, KIRC. (B) Correlation between NUP155 and immune scores in GBM, STES, UCEC, THYM, ESCA, STAD, LUSC, SKCM.


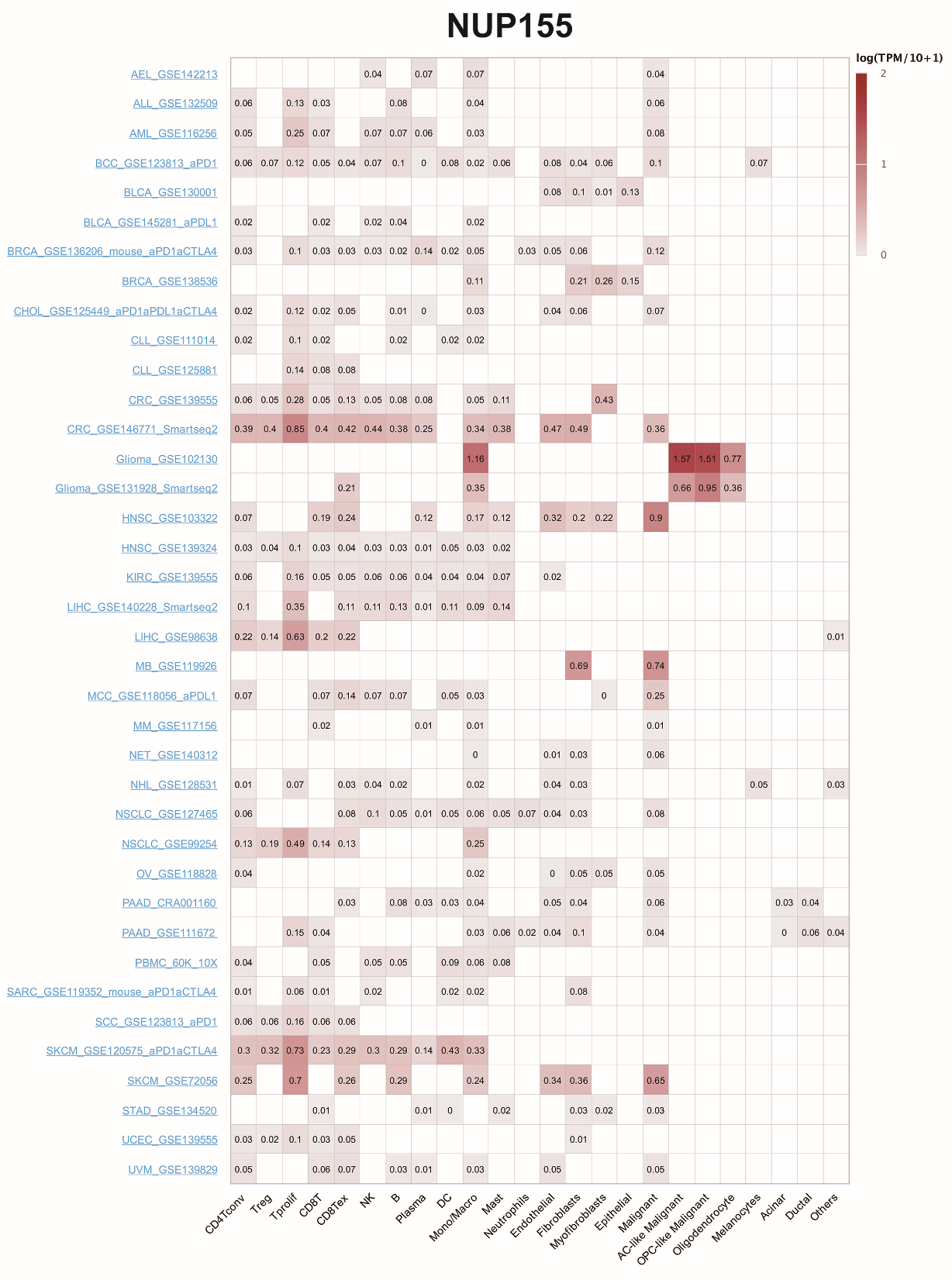


**Supplementary Figure 6:** Association between *NUP155* gene and the TIME in pan-cancer tissues, using the TISCH database.


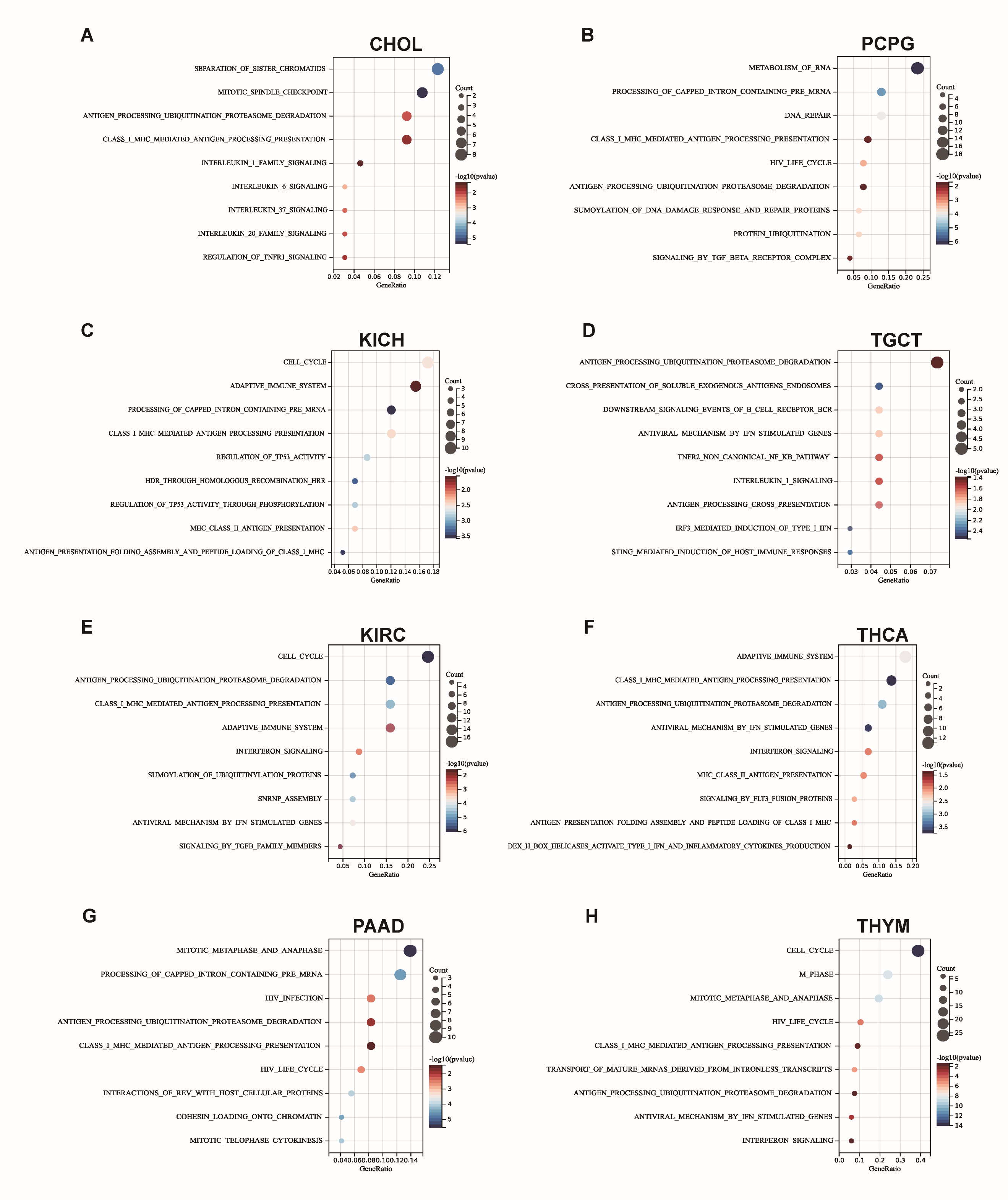


**Supplementary Figure 7:** Reactome functional annotation of *NUP155* in various cancers, including CHOL (A), PCPG (B), KICH (C), TGCT (D), KIRC (E), THCA (F), PAAD (G), and THYM (H).


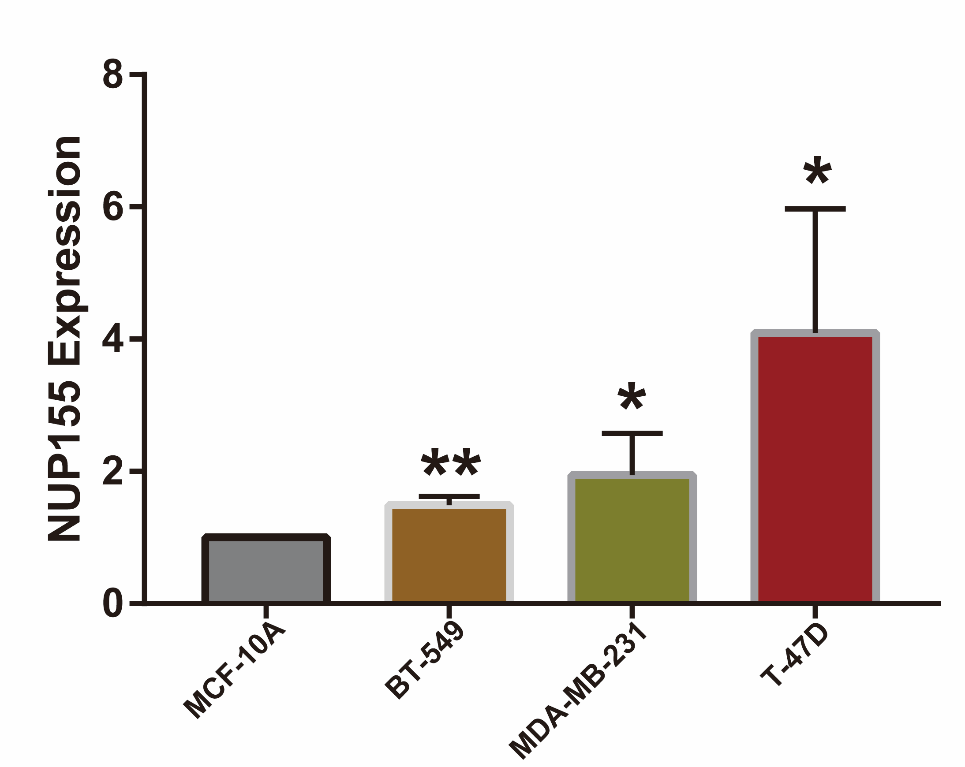


**Supplementary Figure 8：**Relative mRNA expression of *NUP155* in normal breast cell and breast cancer cell lines. *p < 0.05 and **p < 0.01.
